# Discovery of ultrafast myosin, its amino acid sequence, and structural features

**DOI:** 10.1073/pnas.2120962119

**Published:** 2022-02-16

**Authors:** Takeshi Haraguchi, Masanori Tamanaha, Kano Suzuki, Kohei Yoshimura, Takuma Imi, Motoki Tominaga, Hidetoshi Sakayama, Tomoaki Nishiyama, Takeshi Murata, Kohji Ito

**Affiliations:** ^a^Department of Biology, Graduate School of Science, Chiba University, Chiba 263-8522, Japan;; ^b^Department of Chemistry, Graduate School of Science, Chiba University, Chiba 263-8522, Japan;; ^c^Faculty of Education and Integrated Arts and Sciences, Waseda University, Shinjuku-ku, Tokyo 162-8480, Japan;; ^d^Department of Integrative Bioscience and Biomedical Engineering, Graduate School of Science and Engineering, Waseda University, Shinjuku-ku, Tokyo 162-8480, Japan;; ^e^Department of Biology, Graduate School of Science, Kobe University, Nada-ku, Kobe 657-8501, Japan;; ^f^Research Center for Experimental Modeling of Human Disease, Kanazawa University, Kanazawa 920-0934, Japan;; ^g^Membrane Protein Research and Molecular Chirality Research Center, Chiba University, Chiba 263-8522, Japan;; ^h^Structure Biology Research Center, Institute of Materials Structure Science, High Energy Accelerator Research Organization (KEK), Tsukuba 305-0801, Japan

**Keywords:** molecular motor, cytoplasmic streaming, myosin, actin, crystal structure

## Abstract

Cytoplasmic streaming with extremely high velocity (∼70 μm s^−1^) occurs in cells of the characean algae (*Chara*). Because cytoplasmic streaming is caused by myosin XI, it has been suggested that a myosin XI with a velocity of 70 μm s^−1^, the fastest myosin measured so far, exists in *Chara* cells. However, the velocity of the previously cloned *Chara corallina* myosin XI (*Cc*XI) was about 20 μm s^−1^, one-third of the cytoplasmic streaming velocity in *Chara*. Recently, the genome sequence of *Chara braunii* has been published, revealing that this alga has four myosin XI genes. We cloned these four myosin XI (*Cb*XI-1, 2, 3, and 4) and measured their velocities. While the velocities of *Cb*XI-3 and *Cb*XI-4 motor domains (MDs) were similar to that of *Cc*XI MD, the velocities of *Cb*XI-1 and *Cb*XI-2 MDs were 3.2 times and 2.8 times faster than that of *Cc*XI MD, respectively. The velocity of chimeric *Cb*XI-1, a functional, full-length *Cb*XI-1 construct, was 60 μm s^−1^. These results suggest that *Cb*XI-1 and *Cb*XI-2 would be the main contributors to cytoplasmic streaming in *Chara* cells and show that these myosins are ultrafast myosins with a velocity 10 times faster than fast skeletal muscle myosins in animals. We also report an atomic structure (2.8-Å resolution) of myosin XI using X-ray crystallography. Based on this crystal structure and the recently published cryo-electron microscopy structure of acto-myosin XI at low resolution (4.3-Å), it appears that the actin-binding region contributes to the fast movement of *Chara* myosin XI. Mutation experiments of actin-binding surface loops support this hypothesis.

## Significance

It has been suggested for more than 50 y that the fastest myosin in the biological world with a velocity of 70 μm s^−1^ exists in the alga *Chara*, because cytoplasmic streaming with a velocity of 70 μm s^−1^ occurs in *Chara* cells. However, a myosin with that velocity has not yet been identified. In this work, we succeeded in cloning a myosin XI with a velocity of 60 μm s^−1^, which was measured using a chimeric myosin. We also successfully crystallized myosin XI. Structural comparison of various myosins and mutation experiments of actin-binding regions suggests that the central regions that define the fast movement of *Chara* myosin XI are the actin-binding sites.

Myosins are motor proteins that convert chemical energy, ATP, to physical force to move actin filaments. Phylogenetic analyses of myosin motor domain (MD) sequences have shown that there are at least 79 myosin classes, with several subclasses under each class (1). Myosins of different classes and subclasses differ significantly in properties such as velocity, ATPase activity, and duty ratio (the proportion of the ATPase cycle in which the MD remains strongly bound to actin) and perform different intracellular functions (2). The diversity of properties of these classes and subclasses arise from differences in the rates of the binding and dissociation of ATP, ADP, and actin filaments (3).

Plants have two plant-specific myosin classes, myosin VIII and myosin XI. Myosin VIII moves actin filaments at very slow velocities (4) and is involved in endocytosis, cell plate formation, and plasmodesmatal functioning in plants (5–7). Myosin XI produces an intracellular flow known as cytoplasmic streaming in plant cells by moving on actin filaments while binding organelles via its tail domain. Cytoplasmic streaming facilitates the distribution of molecules and vesicles throughout large plant cells (8–12). The velocities of myosin XI are generally high, and the molecule specializes in cytoplasmic streaming. Some cells of characean algae (*Chara*) are very large, being up to 10 cm long and 0.1 cm in diameter. Very fast cytoplasmic streaming, of up to 70 μm s^−1^, is required for the dispersal of molecules and vesicles into the giant *Chara* cells (13).

Based on the velocity of cytoplasmic streaming in *Chara* cells, it has long been suggested that *Chara* has a myosin moving on actin filaments at 70 µm s^−1^ (13–17). This velocity is 10 times faster than the velocity of fast skeletal muscle myosin and the fastest of all myosins measured. A motor protein isolated from *Chara* cells moved actin filaments at 60 μm s^−1^ (18). The development of approaches for cloning this ultrafast myosin is urgently needed. Details of the sequence of the protein and the ability to work with cloned myosin constructs will allow the investigation of the mechanisms that control the myosin velocity and facilitate investigation of the detailed chemical–mechanical conversion mechanism of myosin (19). Kashiyama et al. cloned the complementary DNA (cDNA) of *Chara* myosin from a *Chara corallina* cDNA library by immunoscreening using antibodies against purified *C. corallina* myosin (20). Morimatsu et al. also cloned the cDNA of *Chara* myosin using the same method as that used by Kashiyama et al. (21). The sequences of the MD of myosins cloned by the two groups were identical, and there was a 15 amino acid indel variation in the tail domain, a finding that indicates potential alternative splicing in the tail domain. The *C. corallina* myosin XI (*Cc*XI) contains six isoleucine–glutamine (IQ) motifs, which are light chain–binding sites. It was not possible to express the protein and measure its velocity using the cloned *Cc*XI, because the myosin light chains that bind to the six IQ motifs of *Cc*XI have not been identified. Therefore, the functional expression of *Cc*XI has been carried out using either a *Cc*XI MD construct that did not have the myosin light chain–binding sites (IQ motifs) or chimeric full-length *Cc*XI constructs in which IQ motifs and myosin light chains of *Cc*XI were replaced with those of other myosins. The velocity of *Cc*XI was then estimated from the velocity measured using these constructs. The estimated velocity of *Cc*XI was about 20 µm/s^−1^ or less at 25 °C (10, 22–26), which is less than about one-third of the velocity of cytoplasmic streaming observed in *Chara* cells. Three possibilities have been suggested as to why the velocities of *Cc*XI obtained using the recombinant constructs were different from that expected from cytoplasmic streaming (1). The recombinant *Cc*XI constructs do not have the same IQ motifs and myosin light chains as native *Cc*XI, and this substitution may have affected the velocity (2). *Cc*XI may undergo a posttranslational modification in *Chara* cells, which may increase the velocity of *Cc*XI in cells (3). A myosin XI gene other than *Cc*XI may be present in *Chara* cells, and this myosin XI may be responsible for cytoplasmic streaming with a velocity of 70 µm s^−1^.

Recently, a genome project (*Chara braunii* genome sequencing project, National Center for Biotechnology Information (NCBI) Bio Project ID: PRJDB3348) has been conducted for *C. braunii* (27). *C. braunii* is phylogenetically close to *C. corallina* (28–30), and both species have the same cytoplasmic streaming velocity, 70 µm s^−1^. The *Chara* genome project revealed that the *C. braunii* genome contains four myosin XI genes.

In this study, we cloned the four *C. braunii* myosin XIs and named them *Cb*XI-1, *Cb*XI-2, *Cb*XI-3, and *Cb*XI-4. Phylogenetic analyses indicated that the myosin XIs in *Chara* form a clade in streptophyte myosin XIs, expanded independently from seed plant myosin XIs, and gave rise to the four members in *C. braunii*. *Cb*XI-4 may be an ortholog of *Cc*XI. We show that the velocity of *Cb*XI-1 (60 µm s^−1^) is almost the same as the velocity of cytoplasmic streaming in *Chara* cells, the fastest currently known in the biological world. We also succeeded in crystallizing *Arabidopsis* myosin XI-2 (*At*XI-2), an atomic structure of myosin XI and a valuable comparator for the *Chara* myosin. Structural analyses and mutation experiments suggest that the central regions that define *Chara* myosin XI's fast movement are the actin-binding sites.

## Results

### Phylogenetic Relationships of the Four *Cb*XIs.

**Fig. 1.**
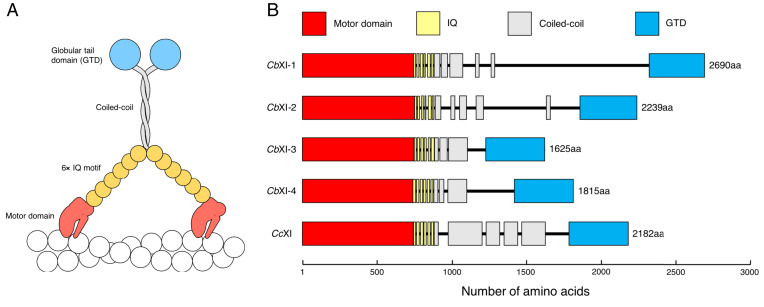
*Cb*XI structure deduced from its amino acid sequence. (*A*) Schematic diagrams of native *Cb*XIs. *Cb*XIs show typical domain structures of myosin XI. They contain an MD with nucleotide- and actin-binding sites, six IQ motifs to which six myosin light chains bind, an α-helical coiled-coil domain leading to dimer formation, and a GTD. (*B*) Domain structures of four *C. braunii* and one *Cc*XIs. Domains and motifs indicated by colored boxes were predicted using the MOTIF Search (https://www.genome.jp/tools/motif/) and COILS programs (58). Black lines are regions that were not recognized as known domains or motif structures. The length of each box and each line is proportional to the number of amino acids occupying each region. Amino acid numbers and sequences and full-length, MD, IQ motifs, coiled-coil, and GTD of *Cb*XI-1, *Cb*XI-2, *Cb*XI-3, *Cb*XI-4, and *Cc*XI are shown in *SI Appendix*, *Supplementary Text*.

Until 2018, the only known myosin sequence in the genus *Chara* was that of *Cc*XI, which was cloned by Kashiyama (20) and Morimatsu (21) independently in 2000. Their results suggested that *Chara* has only one myosin XI gene. However, the *C. braunii* genome project, published in 2018, revealed four myosin XI genes having intact MD: g50407, g48390, g24025, and g48658 (27). We named g50407, g48390, g24025, and g48658 as *Cb*XI-1, *Cb*XI-2, *Cb*XI-3, and *Cb*XI-4, respectively. The originally annotated g48658 (*Cb*XI-4) was truncated at the N-terminal 743 amino acids. The rest of the messenger RNA (mRNA) sequence of *Cb*XI-4 was identified on another scaffold, based on transcriptome assemblies (*SI Appendix*, *Materials and Methods* and accession nos: BR001749 and BR001750 for two isoforms). *Cb*XI-1 differed in the cloned MD sequence (LC641776) from the prediction and the full-length sequence (BR001757) was reconstructed based on the cloned MD sequence. The full-length amino acid sequences of *Cb*XI-1, *Cb*XI-2, *Cb*XI-3, and *Cb*XI-4 are shown in *SI Appendix*, *Supplementary Text*. A schematic diagram of the *Cb*XIs deduced from the amino acid sequences is shown in Fig. 1. *Cb*XIs have typical domain structures of myosin XI: a MD, a neck domain with six IQ motifs to which six myosin light chains bind, a coiled-coil domain for dimer formation, and a globular tail domain (GTD).

**Fig. 2.**
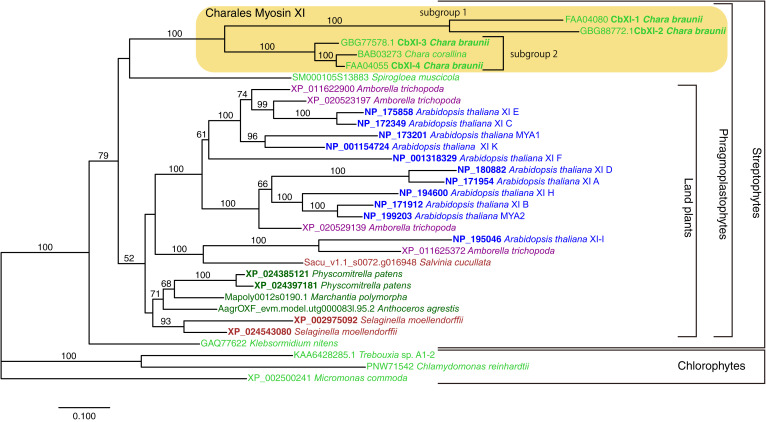
Phylogenetic relationship of green plant myosin XI genes. The phylogenetic tree was constructed using RAxML with -m PROTGAMMALGF option. Amino acid sequences of 32 representative myosin XI genes including five genes from *Chara* were retained in the alignment, and 1,060 sites in conserved regions were used for the analysis. Bootstrap analysis was performed with 1,000 replicates, and the percentage values are indicated on each branch >50%. The horizontal branch lengths are proportional to the estimated number of substitutions per site. Identifiers for *Salvinia cucullata*, *Anthoceros agrestis*, and *Marchantia polymorpha* are from respective genome databases; other identifiers are accession numbers for International Nucleotide Sequence Database Collaboration (INSDC).

We examined the phylogenetic relationships among the myosin XIs from *Chara* and representative green plants (Fig. 2). The phylogenetic tree indicated that streptophyte myosin XIs formed a well-supported clade including genes from *Klebsormidium nitens* and the Phragmoplastophyta, which includes *Chara*, *Spirogloea*, and the land plants. However, the basal relationship within Phragmoplastophyta was not clearly resolved. The four *Cb*XI genes and a *Cc*XI gene formed a well-supported clade (Fig. 2, light-yellow box; Charales myosin XI). Within the Charales myosin XI clade, *Cb*XI-1 and *Cb*XI-2 formed a clade (subgroup 1) sister to the remaining three genes (subgroup 2). *Cb*XI-4 and *Cc*XI formed a clade, and *Cb*XI-3 diverged earlier. The subgroup 1 has notably longer branches compared with subgroup 2 or other green plant myosin XIs. The proteins encoded by these two subgroups 1 genes are apparently larger than the subgroup 2 genes, and especially long are the regions between coiled-coil and GTD domains (Fig. 1*B*).

### Recombinant Constructs of *Cb*XIs.

To clarify the biochemical properties of the four *Cb*XIs, it was necessary to isolate and purify each *Cb*XI. However, it is very difficult to purify active myosins from *Chara* cells, because most of the cell volume of *Chara* cells is occupied by the vacuole, which is rich in proteolytic enzymes, and the cytoplasm is only a small volume (31). Furthermore, it is virtually impossible to purify each of the four myosin XIs with similar molecular properties from *C. braunii* cells. The only way to obtain each of four *Cb*XIs is to express and purify them using a recombinant construct. Using baculovirus expression systems for the functional expression of myosins with IQ motifs, coexpression with myosin light chains that bind to the IQ motifs is required. Calmodulin binds IQ motifs in the myosin neck region and functions as myosin light chains for many animal unconventional myosins, such as myosin I, myosin V, and myosin VI. Analysis of the IQ motif sequences of the animal unconventional myosins, in which calmodulin acts as a light chain, using Pfam database (http://pfam.xfam.org) (32) shows that most of the sequences fall into the typical calmodulin-binding IQ motif (33), especially the first IQ motif (*SI Appendix*, Table S1). On the other hand, the IQ motif sequences of some plant myosin XIs, such as *Cc*XI, *At*XI-A, *At*XI-D, *At*XI-I, and *At*XI-J, are far from the typical calmodulin-binding motif (*SI Appendix*, Table S1). Coexpression of these myosin XIs with calmodulin in insect cells did not yield functional myosin (23, 34). We coexpressed a *Cb*XI-1 construct containing the native IQ motifs with calmodulin in insect cells and tried to purify the construct, but it did not work as predicted from the IQ motifs. It is likely that calmodulin failed to bind to the native IQ motifs of *Cb*XI-1, exposing the hydrophobic region of the IQ motifs and causing the *Cb*XI-1 to aggregate in the cells.

We, therefore, expressed *Cb*XIs using approaches that did not include the sequences of the IQ motifs of *Chara* myosins. We used two types of constructs: *Cb*XI MD and chimeric *Cb*XI (*SI Appendix*, Fig. S1). *Cb*XI MD consists of only the MD of *Cb*XI. Chimeric *Cb*XI consists of the MD of *Cb*XI and six IQ motifs and coiled-coil of *Arabidopsis* XI-F *(At*XI-F). Because calmodulin binds to the six IQ motifs of *At*XI-F as light chains (34) (*SI Appendix*, Table S1), coexpression of the chimeric *Cb*XI and calmodulin would yield functional *Cb*XIs with the same lever arm length as native *Cb*XIs. These constructs were expressed in a baculovirus system and purified by nickel-affinity and FLAG-affinity resins (*SI Appendix*, *Materials and Methods*). The purity and homogeneity were confirmed by SDS-polyacrylamide gel electrophoresis (SDS-PAGE) (*SI Appendix*, Figs. S2 and S3). It was also confirmed by SDS-PAGE that calmodulin bound to the purified chimeric *Cb*XI (*SI Appendix*, Fig. S3).

### Velocities of *Cb*XIs.

**Fig. 3.**
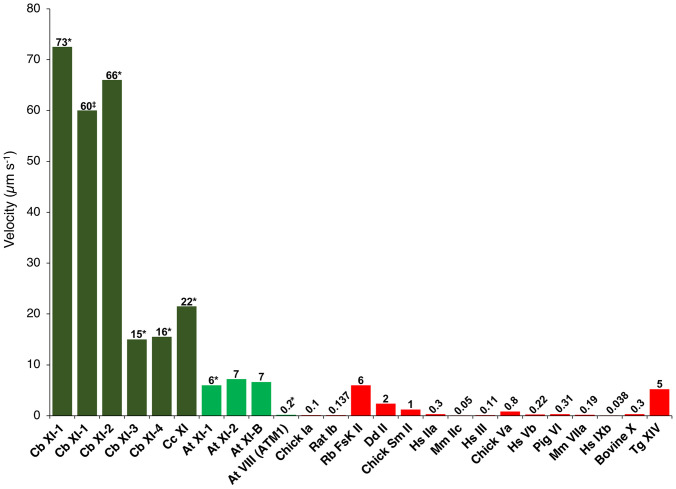
Velocities of various classes of myosins. *Cb*XI: *Chara braunii* myosin XI (this paper), *Cc*XI: *Chara corallina* myosin XI (23, 24), *At* XI: *Arabidopsis thaliana* myosin XI (10, 34), *At* VIII: *Arabidopsis thaliana* myosin VIII (4), Chick Ia: Chicken myosin Ia (59), Rat Ib: Rat myosin Ib (60), Rb FskII: Rabbit fast skeletal myosin II (61), *Dd* II: *Dictyostelium discoideum* myosin II (62), Chick Sm II: Chicken gizzard smooth muscle myosin II (63), *Hs* IIa: *Homo sapiens* myosin IIa (64), Mm IIc, *Mus musculus* myosin IIc (65), *Hs* III: *Homo sapiens* myosin III (66), Chick V: Chicken myosin Va (67), *Hs* Vb: *Homo sapiens* myosin Vb (68), Pig VI: Pig myosin VI (69), Mm VIIa: *Mus musculus* myosin VIIa (70), Hs IXb: Homo sapiens myosin IXb (71), Bovine X: Bovine myosin X (72), and Tg XIV: *Toxoplasma gondii* myosin XIV (73). *Estimated value from the velocity of MD constructs. ^‡^Value of the chimeric *Cb*XI-1construct.

The sliding velocities of actin filaments by *Cb*XI MD were measured using an antibody-based version of the in vitro motility assay at 25 °C. *Cb*XI-3 MD and *Cb*XI-4 MD, belonging to the subgroup 2, moved actin filaments at velocities of 3.0 ± 0.2 and 3.1 ± 0.3 μm s^−1^, respectively, which were similar to that of *Cc*XI MD (23, 24). The velocities of *Cb*XI-1 MD and *Cb*XI-2 MD, belonging to the subgroup 1, were 15 ± 0.7 μm s^−1^ and 13 ± 0.6 μm s^−1^, respectively, which were about threefold faster than that of *Cc*XI MD (23, 24) (Table 1). Actin velocities generated by myosins are approximately proportional to the lever arm length of myosin if the motor region is the same (35, 36). We have previously shown that this relationship between lever arm length and actin velocities by myosin generally holds for myosin XIs. Based on the crystal structure of myosin V with IQ motifs (37) (Protein Data Bank [PDB]: 2IX7), an ortholog of myosin XI, it is estimated that the lever arm length of myosin XI MD is 3.5 nm, which is 1/6.6 that of full-length myosin XI (native myosin XI) containing six IQ motifs (23 nm). The velocity of myosin XI MD was one-fifth that of full-length myosin XI (34). Therefore, multiplying the velocity of myosin XI MD by a factor of 5 gives the velocity of native (full-length) myosin XI. The estimated velocities of native (full-length) *Cb*XI-1 and *Cb*XI-2 were 73 µm s^−1^ (14.5 μm/s^−1^ × 5) and 66 µm s^−1^ (13.2 μm s^−1^ × 5), respectively (Fig. 3 and Table 1). The estimated velocities of *Cb*XI-1 and *Cb*XI-2 are therefore almost the same as the cytoplasmic streaming velocity in members of the genus *Chara* (*C. corallina* and *C. braunii*), 70 µm s^−1^ (13, 17).

**Table 1.** *V*_max_ and *K*_app_ of actin-activated ATPase activity and actin-sliding velocity of *Chara* myosin*

	*Cb*XI-1	*Cb*XI-2	*Cb*XI-3	*Cb*XI-4	*Cc*XI^†^
* V*_max_ (Pi/s^−1^/head^−1^)	410	200	260	230	580
* K*_app_ (µM)	46	45	15	8.7	23
Velocity of MD (μm/s^−1^)^‡^	15 ± 0.7	13 ± 0.6	3.0 ± 0.2	3.1 ± 0.3	4.7 ± 0.3
Estimated velocity of full-length myosin^§^ (μm/s^−1^)	73	66	15	16	24
Velocity of chimeric full-length myosin (μm/s^−1^)	60 ± 4.1^¶^	ND	ND	ND	16 ± 0.9^#^
Duty ratio^‖^ (%)	9.6	5.4	30	26	43

*Measured at 25 °C.

^†^*V*_max_, *K*_app_, velocity of *Cc*XI MD (24), and velocity of the chimeric full-length *Cc*XI (10).

^‡^mean ± SD; *n* = 30.

^§^Estimated values from the velocity of the MD constructs, which are calculated by the method described in ref. 34.

^¶^Value of the chimeric *Cb*XI-1, mean ± SD, and *n* = 30.

^#^Value of the chimeric *Cc*XI (10), mean ± SD, and *n* = 30.

‖Calculated by the *V*_max_ values of the actin-activated ATPase activities and the velocities of MD as described in ref. 34.

To examine the velocity of *Cb*XI-1 using a construct that is structurally similar to the native *Cb*XI-1, we used chimeric *Cb*XI-1 (*SI Appendix*, Fig. S1). The velocity of chimeric *Cb*XI-1 was 60 ± 4.1 μm s^−1^ (Table 1, and Movie S1). While this velocity was somewhat less than the velocity estimated from the velocity of *Cb*XI-1 MD and the cytoplasmic streaming velocity of the *Chara* cells (70 μm s^−1^), the values are almost the same as the reported myosin velocity purified from *Chara* cells (60 μm s^−1^) (18). The lower velocity of the chimeric *Cb*XI-1 may be due to improper linkage between *Cb*XI-1MD and the IQ motifs of *At*XI-F. Another possible reason for the lower velocity of the chimeric *Cb*XI-1 may be due to the difference in the environment between *Chara* cells and in vitro motility assay. In the in vitro motility assay, actin filaments move on myosins coated randomly on flat surfaces. In contrast, in *Chara* cells, vesicle-associated myosins move on polarized actin filaments (38). Because myosins moving on polarized actin filaments have been reported to be faster (39), the chimeric *Cb*XI-1 may also have a velocity of 70 μm s^−1^ if it moves in *Chara* cells. The results in this study suggest that *Cb*XI-1 and *Cb*XI-2 would be the myosins causing cytoplasmic streaming in *C.braunii* and demonstrate that *Cb*XI-1 with a velocity of 60 µm s^−1^ is the fastest myosin yet measured among all organisms (Fig. 3).

### ATPase Activities.

The ATPase activities of *Cb*XI-1, *Cb*XI-2, *Cb*XI-3, and *Cb*XI-4 MDs were plotted as a function of actin concentration and fit to the Michaels–Menten equation to determine the maximum rate of ATP turnover (*V*_max_) and the actin concentration at which the ATPase rates were one-half of the maximal rate (*K*_app_). The *V*_max_ and *K*_app_ values of *Cb*XI-1 MD were 410 Pi s^−1^head^−1^ and 46 μM, respectively (Table 1). This *V*_max_ value is similar to that of *Cc*XI MD (22, 23), although the actin velocity of *Cb*XI-1 MD is three times faster than that of *Cc*XI MD. The *V*_max_ of actin-activated ATPase activities was not correlated with actin velocity among the four *Cb*XIs. This discrepancy may arise because the rate-limiting step of *V*_max_ of actin-activated ATPase activities (phosphate dissociation from actin–myosin–ADP·Pi complex) and the rate-limiting step of actin velocity (ADP dissociation from actin–myosin–ADP complex) are different (23).

### Crystal Structure of the Myosin XI MD.

To investigate the molecular mechanism of the ultrafast movement of *Cb*XI-1, we tried to crystallize *Cb*XI-1 MD. However, we were unsuccessful, probably because *Cb*XI-1 MD is unstable and prone to semi-denaturation. ATPase activity of *Cb*XI-1 tends to drop in a relatively short time compared with other myosins, which makes it difficult to obtain the crystal structure. Such instability of ATPase activity was observed for all *Chara* myosins. Although class XI myosin is the fastest myosin class in the myosin superfamily (Fig. 3), the atomic structure of the class XI myosin MD has yet to be solved. Therefore, in this study, we decided to clarify the structural features responsible for the high velocity of class XI myosins by crystallographic analysis of other class XI myosins. We chose *Arabidopsis* myosin MYA2 (*At*XI-2) as the crystallization target, because *At*XI-2 has a standard velocity among class XI myosins, it is faster than most animal myosins (Fig. 3) (34), and its ATPase activity is more stable than those of *Chara* myosins. *At*XI-2 has an amino acid sequence relatively similar to that of *Cb*XI-1 MD, having 63% identity and 87% similarity.

**Fig. 4.**
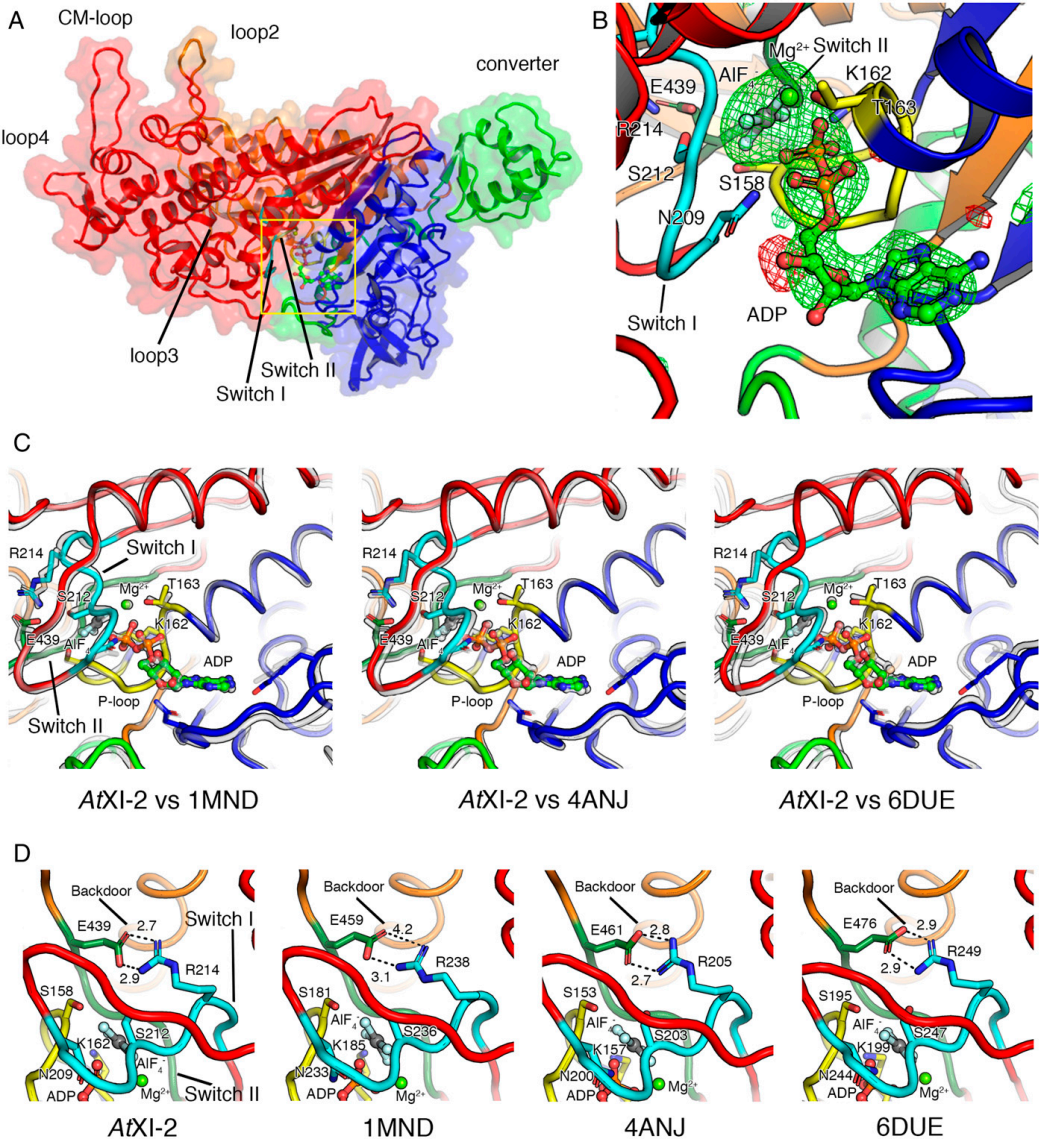
Crystal structure of *At*XI-2 MD bound with ADP and AlF_4_^−^. Upper 50k, Lower 50k, N-terminal, and converter subdomains are colored in red, orange, blue, and green, respectively. Switch I and switch II in the Upper 50k domain are shown in cyan and moss green. (*A*) Four subdomains (Upper 50k, Lower 50k, N-terminal, and converter subdomains) and four actin-coupled surface loops (loop2, loop3, loop4, and CM loop) in *At*XI-2 MD are shown. (*B*) The nucleotide-binding region of *At*XI-2 MD. The |Fo|-|Fc| map calculated without ADP:Mg^2+^ and AlF_4_^−^ at the binding pocket contoured at 4.0 sigma are shown in red (negative) and green (positive). (*C*) Comparison of the nucleotide-binding regions in various myosins (gray) bound to ADP and AlF_4_^−^ shows that the positions of switch I and switch II are almost the same between different classes of myosins. (*D*) Comparison of the size of the backdoor for the Pi release in various myosins bound to ADP and AlF_4_^−^ shows that the size of the backdoor, which is the space between E439 and R214, is almost the same. *Dictyostelium* myosin II (PDB: 1MND), Pig myosin VI (PDB: 4ANJ), and *Toxoplasma gondii* myosin XIV (PDB: 6DUE).

We succeeded in solving the crystal structure of *At*XI-2 MD bound with ADP and AlF_4_^−^ at 2.8-Å resolution, which is the first atomic structure of the class XI myosin MD (Fig. 4 *A* and *B*, and *SI Appendix*, Table S2). Fig. 4 *C* and *D* show comparisons of the structure of ADP·AlF_4_^−^–bound *At*XI-2 with the structures of other ADP·AlF_4_^−^–bound classes of myosins. Although there are some deviations in the position of the main chain, the nucleotide interaction region including switch I and switch II is almost identical (Fig. 4*C*). The backdoor size for phosphate dissociation of *At*XI-2 was also very similar to those of myosins of other classes (Fig. 4*D*). Thus, the structure near the nucleotide-binding region of myosin XI was not markedly different from those of myosins of other classes (myosin II, myosin VI, and myosin XIV).

**Fig. 5.**
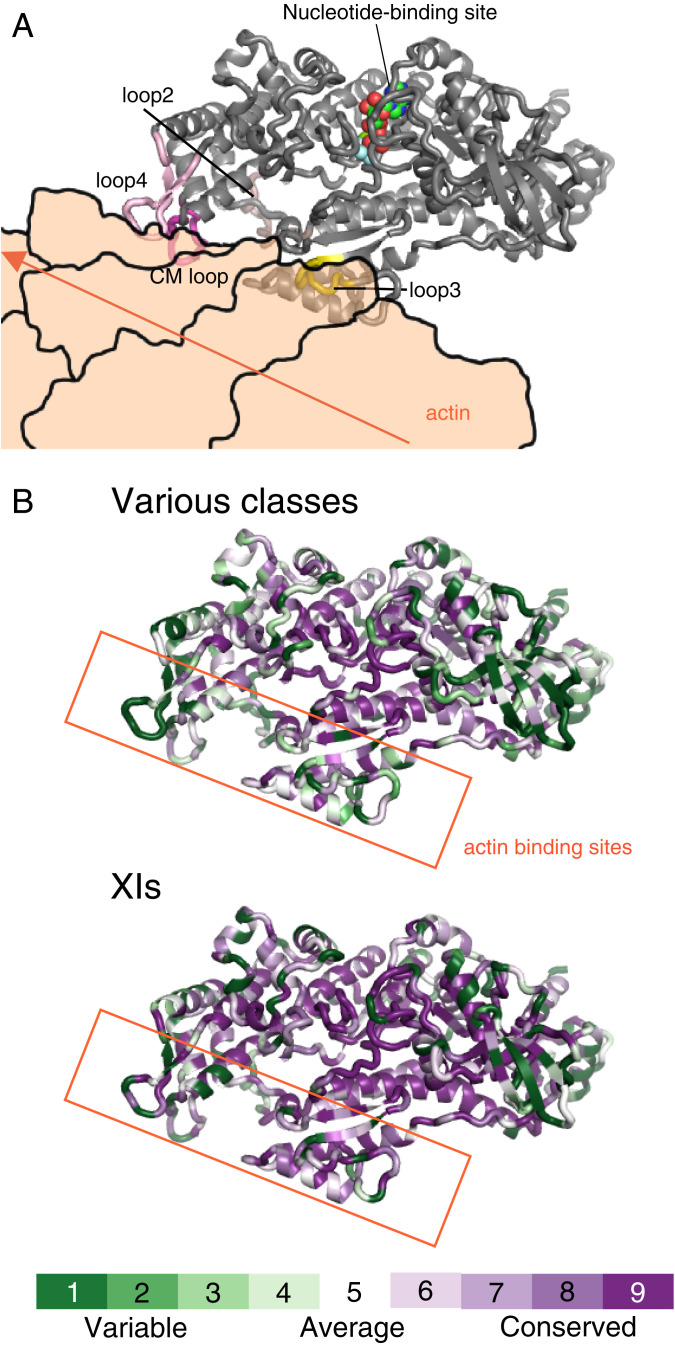
Actin-binding region with high amino acid diversity among myosins. (*A*) Docking model of *A*tXI-2 MD and actin. This model was created by replacing myosin X in 5KG8 (Rigor myosin X cocomplex with an actin filament) with *At*XI-2 MD. The actin in 5KG8 was replaced by 6BNO (structure of bare actin filament). (*B*) Heat map visualization of *A*tXI-2 MD showing amino acid conservation and diversity, generated using ConSurf (https://consurf.tau.ac.il/). The conservation score is calculated using the Maximum Likelihood paradigm. The amino acids of myosins are colored by conservation score ranging from green (1, most variable) to purple (9, most conserved residues), as shown in the color legend. The rate was changed to 1 for residues with four or fewer sequence overlaps in the alignment. Various classes showing amino acid comparison of 10 classes of myosins: class I (human Ic and Rat Ib), class II (*Dictyostelium* II, chicken fast skeletal muscle, chicken smooth muscle, and rabbit skeletal muscle), class V (chicken Va and human Vb), class VI (pig VI), class VII (*Drosophila* VIIa and mouse VIIb), class VIII (*Arabidopsis* VIIIa and *Arabidopsis* VIIIb), class IX (human IXa and human IXb), class X (cow X), class XI (*Arabidopsis* XI-2, *Chara corallina* XI, and *Chara braunii* XI-1), and class XIV (*Toxoplasma* XIV). XIs showing the amino acid comparison of 18 myosins belonging to class XI: *Arabidopsis* XI-1, *Arabidopsis* XI-2, *Arabidopsis* XI-A, *Arabidopsis* XI-B, *Arabidopsis* XI-C, *Arabidopsis* XI-D, *Arabidopsis* XI-E, *Arabidopsis* XI-F, *Arabidopsis* XI-G, *Arabidopsis* XI-H, *Arabidopsis* XI-I, *Arabidopsis* XI-J, *Arabidopsis* XI-K, *Chara corallina* XI, *Chara braunii* XI-1, *Chara braunii* XI-2, *Chara braunii* XI-3, and *Chara braunii* XI-4.

Myosins of different classes have significantly different motor properties, such as velocity, ATPase activity, and duty ratio. Even within the same class, the motor properties are different for each myosin. The differences in the properties of each myosin motor are due to differences in the MD's amino acid sequence. Therefore, we investigated the structural regions in the MD with large variations in amino acid sequence among various myosin classes (Fig. 5). A comparison of the amino acids of different myosins showed that the central part of the MD is highly conserved, and these regions are responsible for basic chemical–mechanical energy conversion. In addition to the Src homology 3 region of the N-terminal subdomain, the actin-binding regions of the upper 50k and lower 50k subdomains have high amino acid diversity among various myosin classes (Fig. 5*B*, various classes and *SI Appendix*, Fig. S4). The same tendency was observed among myosin XIs, although weaker than among various myosin classes (Fig. 5*B*, XIs). These regions would be responsible for the diversity of motor properties among myosins.

The amino acid sequences of *Cb*XI-1 MD and *Cc*XI MD are similar (identity and similarity are 65% and 89%, respectively). Thus, we built a homology model of acto-*Cb*XI-1 rigor structure based on the recently reported acto-*Cc*XI rigor structure (PDB: 7KCH) (26). We then compared the actin-binding modes of six myosins (*Cb*XI-1, *Cc*XI, NM2c, Myosin VI, Myosin 1b, and Pf MyoA) using acto-myosin rigor structures (*SI Appendix*, Fig. S4). Footprint analyses of loop 4 and CM loop of six myosins show that the amino acid residues of actin and myosin in the binding region differed in different classes of myosin but also between *Cc*XI and *Cb*XI-1 in the same class (*SI Appendix*, Table S3). These results indicate that actin-binding modes differ depending on myosins, even for the same class of myosins. Differences in the actin-binding modes may give rise to the diversity in myosin properties such as velocity and ATP activity.

### Velocities of Actin-Binding Surface Loop Mutants of *Cc*XI.

The actin-binding region of myosin consists mainly of four actin-binding surface loops of variable length and composition (loop 2, loop 3, loop 4, and CM loop) and a relatively conserved helix-turn-helix of the Lower 50k subdomain (40–43). Many studies have been conducted on the effects of these actin-binding surface loops on the motor activities using various myosins and have shown that these loops affect the actin-activated ATPase and the velocity (24, 44–51). To investigate the relationship between the diversity of actin-binding regions of myosin and the diversity of myosin velocities among *Chara* myosins, we changed loop 2, loop 3, loop 4, and CM loop of *Cc*XI (subgroup 2) MD to those of *Cb*XI-1 (subgroup 1) and examined whether the velocities of *Cc*XI MD with the loops of *Cb*XI-1 would increase. When the sequences of these four loops were compared among *Chara* myosins, loop 3 sequences were the same except for the amino acid at the C terminus, but loop 2, loop 4, and the CM loop differed variously in length and composition among *Chara* myosins (Table 2). The velocity of *Cc*XI MD with loop 3 of *Cb*XI-1, in which loop 3 of *Cc*XI was replaced with loop 3 of *Cb*XI-1, was almost the same as that of *C*cXI MD. On the other hand, the velocities of *Cc*XI MD with loop 2 of *Cb*XI-1, *Cc*XI MD with loop 4 of *Cb*XI-1, and *Cc*XI MD with CM loop of *Cb*XI-1 were 1.3, 1.1, and 1.3 times higher than that of *Cc*XI MD, respectively. Furthermore, *Cc*XI MD with double mutation of loop 2 and CM loop showed an even-more-significant increase in velocity (1.4-fold) (Table 3). These results indicate that half of the difference in velocity between *Cc*XI and *Cb*XI-1 (about threefold) was due to the differences in the actin-binding surface loops between *Cc*XI and *Cb*XI-1 and that the diversity of the actin-binding region accounts for the diversity of the myosin velocity.

**Table 2.** Loop 2, loop 3, loop 4, and CM loop sequences of *Chara* myosins

Myosin (subgroup)	Loops	Length	Net charge	Amino acid sequence
*Cb*XI-1 (Subgroup 1)	Loop 2	23	1	YPPP*EE*P**K**QGG**K**GGG**K**SSFSSIG
	Loop 3	6	5	**KHK**F**KK**
	Loop 4	15	−2	*D*F**K**PG**K***E*A*D*SSQLA*D*
	CM loop	15	2	Q**R**IMVT**R**G*E*AIT**K**LL
*Cb*XI-2 (Subgroup 1)	Loop 2	25	1	YPPPP*EE*PKQGGG**K**GGS**K**SSFSSIG
	Loop 3	6	5	**KHK**F**KK**
	Loop 4	15	−2	*E*FAPG**K***D*A*D*SS**K**IA*D*
	CM loop	15	3	Q**R**VMMTGT*E***K**I**KK**LL
*Cb*XI-3 (Subgroup 2)	Loop 2	17	0	FPP*DE*GT**K**APS**K**FASIG
	Loop 3	6	4	**R**P**K**F**KR**
	Loop 4	14	−3	*E*FSTGAS*E*AS*E*VSS
	CM loop	16	3	T**R**IM**R**AS**R**T*E*SIT**K**IL
*Cb*XI-4 (Subgroup 2)	Loop 2	17	0	FPL*DE*GA**K**APS**K**FMSIG
	Loop 3	6	5	**KHK**F**KR**
	Loop 4	14	−4	*E*FNSG*E*S*E*AS*E*VST
	CM loop	16	3	T**R**IM**K**AT**R**T*E*SIT**K**IL
*Cc*XI (Subgroup 2)	Loop 2	17	0	FPA*DE*GT**K**APS**K**FMSIG
	Loop 3	6	5	**KHK**F**KR**
	Loop 4	14	−5	*E*F*D*SG*E*S*D*AS*E*VST
	CM loop	16	3	T**R**IM**K**AT**R**T*E*SIT**K**IL

Acidic and basic residues are highlighted in bold and italic, respectively.

**Table 3.** Amino acid sequence and velocity of *Cc*XI MD (wild type) and *Cc*XI MD mutants with actin-binding surface loops of *Cc*XI^†^

Myosin	Loops	Length	Net charge	Amino acid sequence	Velocity(µm/s^−1^)
*Cc*XI MD (wild type)	Loop 2	17	0	FPA*DE*GT**K**APS**K**FMSIG	4.3 ± 0.6
	Loop 3	6	5	**KHK**F**KR**^‡^	
	Loop 4	14	−5	*E*F*D*SG*E*S*D*AS*E*VST	
	CM loop	16	3	T**R**IM**K**AT**R**T*E*SIT**K**IL	
*Cc*XI MD with Loop 2 of *Cb*XI-1	Loop 2	23	1	YPPP*EE*P**K**QGG**K**GGG**K**SSFSSIG	5.5 ± 0.5*
	Loop 3	(same as *Cc*XI)			
	Loop 4	(same as *Cc*XI)			
	CM loop	(same as *Cc*XI)			
*Cc*XI MD with Loop 3 of *Cb*XI-1	Loop 2	(same as *Cc*XI)			4.1 ± 0.4
	Loop 3	6	5	**KHK**F**KK**^2^	
	Loop 4	(same as *Cc*XI)			
	CM loop	(same as *Cc*XI)			
*Cc*XI MD with Loop 4 of *Cb*XI-1	Loop 2	(same as *Cc*XI)			4.9 ± 0.3*
	Loop 3	(same as *Cc*XI)			
	Loop 4	15	−2	*D*F**K**PG**K***E*A*D*SSQLA*D*	
	CM loop	(same as *Cc*XI)			
*Cc*XI MD with CM loop of *Cb*XI-1	Loop 2	(same as *Cc*XI)			5.4 ± 0.3*
	Loop 3	(same as *Cc*XI)			
	Loop 4	(same as *Cc*XI)			
	CM loop	15	2	Q**R**IMVT**R**G*E*AIT**K**LL	
*Cc*XI MD with Loop 2 and CM loop of *Cb*XI-1	Loop 2	23	1	YPPP*EE*P**K**QGG**K**GGG**K**SSFSSIG	6.1 ± 0.2*
	Loop 3	(same as *Cc*XI)			
	Loop 4	(same as *Cc*XI)			
	CM loop	15	2	Q**R**IMVT**R**G*E*AIT**K**LL	

Acidic and basic residues are highlighted in bold and italic, respectively. *P < 0.001 by Student’s *t* test compared with *Cc*XI MD.

^†^Measured at 25 °C, mean ± SD, and *n* = 30.

^‡^Loop 3 sequences of *Cc*XI and *Cb*XI-1 are almost identical, so the only different amino acid residue of loop 3 is underlined.

## Discussion

In this work, we successfully cloned the fastest myosin currently known in the biological world, *Cb*XI-1 with an expected velocity of 60 µm s^−1^. We also succeeded in solving the atomic crystal structure of the MD of class XI myosin. The amino acid sequence features of *Cb*XI-1, mutation experiments, and the crystal structure of class XI myosin indicate that the actin-binding sites are crucial for defining the myosin velocity.

The myosin superfamily currently has 79 classes, each containing several subclasses (1). Of the 79 myosin classes, 17 classes are found in animals and two are found in green plants. Other myosin classes are found in fungi and Protista. The velocities and ATPase activities of most of the 19 myosin classes present in vertebrates and plants have been measured. The velocities of myosins differ greatly depending on the class and subclass. Although skeletal muscle myosin II is exceptionally fast, most animal myosin has a velocity of less than 1 μm s^−1^. In contrast, plant-specific class XI myosins have velocities of 5 µm s^−1^ or higher (Fig. 3). The high velocity of class XI myosins is thought to be related to the plant-specific role; the main role of class XI myosins is to drive cytoplasmic streaming, whereas the role of most animal myosins is the generation of force. The force generated by class XI myosin is smaller than that produced by animal myosins (52). Because plant cytoplasmic streaming is caused by myosin XI-bound organelle movement along actin filaments, the velocity of cytoplasmic streaming in plants is thought to be approximately equal to the velocity of the class XI myosin of that plant. Based on the cytoplasmic streaming velocity in the aquatic genus *Chara*, it has long been proposed that *Chara* possesses a myosin with a velocity of 70 μm s^−1^ (13–18), but the myosin responsible for this had not been identified. This work revealed that *Cb*XI-1 and *Cb*XI-2 are the sought-after fast myosins.

The *Chara* myosin XIs have expanded independently of the expansion of the land plants (53), resulting in four genes. *Cb*XI-1 and *Cb*XI-2, the members of subgroup 1, form a clade having a longer branch than that of a clade formed by *Cb*XI-3, *Cb*XI-4, and *Cc*XI, which are in subgroup 2 (Fig. 2). Because *Cb*XI-4 and *Cc*XI form a clade, they are putative orthologs. The longer branch lengths in the *Cb*XI-1 and *Cb*XI-2 clade imply the action of adaptive evolution to increase the velocity, as they have fastest movement and do not seem to have lost any functionality. The genome project found that *Cb*XI-1 is the most-highly expressed myosin XI in the whole plant (*SI Appendix*, Table S4) (27). Thus, *Cb*XI-1 appears to be the main contributor to cytoplasmic streaming in *C. braunii*. *Cb*XI-2, which has almost the same velocity as *Cb*XI-1, would provide redundancy of the function of *Cb*XI-1.

We estimated the duty ratio of *Cb*XI myosins from the *V*_max_ values of the actin-activated ATPase activities and the velocities of MD (Table 1). The duty ratios of subgroup 2 myosins are much higher than those of subgroup 1. In order for a myosin-bound vesicle to remain associated with actin filaments and to move continuously along the actin filaments, at least one of the myosin MDs on the vesicle must always be strongly bound to the actin filaments. Therefore, the reciprocal of the duty ratio is the lowest number of MDs on a vesicle required for continuous movements of the myosin-bound vesicle on the actin filaments. Subgroup 2 myosins, which have a high duty ratio, can transport vesicles with fewer myosin MDs than can subgroup 1 myosins. Subgroup 2 myosins may function in small vesicle transport, in which the number of bound myosins is limited.

We have obtained a crystal structure of class XI myosin at 2.8-Å resolution, which is the fastest of all the measured myosin classes. The structure of class XI myosin, *At*XI-2, near the nucleotide-binding region was similar to those of other classes of myosins: *Dictyostelium* myosin II, pig myosin VI, and *Toxoplasma* myosin XIV (Fig. 4 *C* and *D*). This finding is consistent with a previous report that the central part of the myosin structure is similar among different classes of myosins, even though their velocities and ATPases are very different (54). On the other hand, there is considerable diversity among the amino acids in the actin-binding sites of myosins, both among the myosin classes and among myosin XIs (Fig. 5). Most recently, the rigor structure (postpower stroke and nonnucleotide state) of acto-*Cc*XI MD determined by cryo-electron microscopy at 4.3-Å resolution was reported (26). Because the amino acid sequences of *Cc*XI MD and *Cb*XI-1 MD are similar, we created a homology model of acto-*Cb*XI-1 MD based on the reported structure of the acto-*Cc*XI MD. Comparing acto-*Cc*XI and acto-*Cb*XI-1, the binding modes of *Cc*XI and *Cb*XI-1 to actin was different (*SI Appendix*, Fig. S4). Footprint analyses showed that the amino acids in the actin-binding sites of loop 4 and CM loop differed between *Cc*XI and *Cb*XI. In addition, the amino acids of actin to which these loops bind are also different between *Cc*XI and *Cb*XI (*SI Appendix*, Table S3). In order to investigate the relationship between the differences in the actin-binding regions and the differences in the myosin velocity, we made *Cc*XI MD with loop 4 of *Cb*XI-1 and *Cc*XI MD with CM loop of *Cb*XI-1. The velocities of *Cc*XI MD with loop 4 of *Cb*XI-1 and *Cc*XI MD with CM loop of *Cb*XI-1 were higher than that of *Cc*XI MD (Table 3).

The velocity of *Cc*XI with loop 2 of *Cb*XI-1 was also higher than that of *Cc*XI (Table 3). It is not possible to determine from the acto-MD structure whether the actin-binding mode of *Cc*XI loop 2 is different from that of *Cb*XI-1 loop 2, because loop 2 was not visible in the reported acto-*Cc*XI structure (26). Loop 2 of *Cb*XI-1 (subgroup 1) is characterized by three consecutive proline and five glycine clusters (Table 2), which are not present in loop 2 of subgroup 2 of *Chara* myosins, such as *Cc*XI. Proline and glycine clusters are known to disrupt the secondary structure (55, 56), so loop 2 of *Cb*XI-1 with this feature would be a completely free loop structure, resulting in increased flexibility. The increased flexibility of loop 2 may allow for fast ADP dissociation from acto-myosin, which increases the myosin velocity.

The difference in velocity between *Cc*XI and *Cb*XI-1 was about threefold (Table 1). About half of this difference was accounted for by the difference in the amino acid sequence of the actin-binding surface loops between *Cc*XI and *Cb*XI-1, because the velocity of *Cc*XI with loop 2 and CM loop of *Cb*XI-1 was 1.4-fold higher than that of *Cc*XI (Table 3). Actin-binding sites other than loops may also be involved in the variation of actin velocity. The L50k subdomain, one of the most-important actin-binding sites and constituting the tertiary structure, shows diversity among myosin classes (Fig. 5). This region may be responsible for the diversity of myosin properties among myosin classes. However, because mutations in the tertiary structure can disrupt the overall structure, mutation experiments on the L50k subdomain may be difficult. A recent paper also reported that the amino acids of actin and myosin in the acto-myosin binding regions vary depending on the type of myosins, similar to our present work (42). Kinetic data from various myosin classes also suggest that actin-binding sites are crucial for the diversity of velocities among the classes and subclasses of myosins. The ADP dissociation rate from the myosin–ADP complex in the absence of actin is nearly identical among myosins with different velocities. In contrast, the ADP dissociation rate from the actin–myosin–ADP complex, the rate-limiting step in myosin velocity, differs depending on the classes and subclasses of myosins (23). Different classes and subclasses of myosins bind to actin in somewhat different ways, which probably cause different changes in the nucleotide-binding site through communication between the actin- and the nucleotide-binging sites within the MD, resulting in different kinetics.

*At*XI-2 MD and *Cc*XI MD are similar in the structures and the amino acid sequences (identity and similarity are 63% and 91%, respectively). Therefore, we estimated the angular change of the converter domain during the power stroke of *Cc*XI using *At*XI-2·ADP (prepower stroke) and *Cc*XI (rigor) structures. The estimated angular angle of *Cc*XI is 85 degrees, which is similar to those of pig myosin VI, Scallop myosin II, and Plasmodium myosin XIV (*SI Appendix*, Fig. S5). This result suggests that the magnitude of the angular change in the converter domain is not related to the fast velocity of class XI myosin.

Detailed kinetics analyses and single-molecule assays using the fastest myosin, *Cb*XI-1, would provide further insights into the detailed chemical–mechanical conversion mechanism of myosin. The gene for *Cc*XI, the fast myosin, has contributed to the development of nanomachines (25, 26) and the enhancement of plant growth (10, 57). The gene for *Cb*XI-1, the ultrafast myosin, will greatly contribute to a variety of research and development.

## Materials and Methods

### RNA Extraction.

Thalli of strain S276 were harvested in soil-water medium for the Charales (SWC-3) (27). Further details are described in *SI Appendix*, *Materials and Methods*.

### Cloning of MD of *C. braunii* Myosins.

cDNA of *Cb*XI-1 (g50407), *Cb*XI-2 (g48390), *Cb*XI-3 (g24025), and *Cb*XI-4 (g48658) MDs were amplified from total RNA of *C.braunii*. Further details are described in *SI Appendix*, *Materials and Methods*.

### Identification of Full-Length Sequence of *Cb*XI-4.

Detailed information is described in *SI Appendix*, *Materials and Methods*.

### Protein Engineering, Expression, and Purification.

Myosins were expressed using a baculovirus expression system and purified using Ni-affinity and FLAG-affinity resins as previously described (4, 23, 24). Further details are described in *SI Appendix*, *Materials and Methods*.

### ATPase Activity.

ATPase activities were determined by measuring released phosphate as previously described (22). The reaction mixtures for the assay of actin-activated Mg^2+^-ATPase activity contained were done in 25 mM KCl, 4 mM MgCl_2_, 25 mM Hepes-KOH (pH 7.4), 2 mM ATP, 1 mM dithiothreitol (DTT), and 1 mg/mL bovine serum albumin and at 25 °C and 0.125 to 4 mg/mL F-actin. Values of ATPase activities are averages of two measurements on two independent preparations.

### In Vitro Motility Assay.

The velocity was measured using an anti-Myc antibody-based version of the in vitro actin filament-gliding assay as previously described (23). The velocity of actin filaments was measured in 150 mM KCl, 4 mM MgCl_2_, 25 mM Hepes-KOH (pH 7.4), 2 mM ATP, 10 mM DTT, and oxygen scavenger system (120 µg/mL glucose oxidase, 12.8 mM glucose, and 20 µg/mL catalase) at 25 °C. Average sliding velocities are determined by measuring the displacements of actin filaments. Values of actin velocities are averages of 30 measurements from two independent preparations.

### Crystallization and Data Collection.

Detailed information is described in SI Appendix, Materials and Methods.

### The Phylogenetic Tree of *Chara* Myosin XIs and the Whole of Myosin XIs.

Detailed information is described in *SI Appendix*, *Materials and Methods*.

## Supplementary Material

Supplementary File

Supplementary File

## Data Availability

Full-length nucleotide sequence, atomic coordinate, and structure factor data have been deposited in DNA Data Bank of Japan (DDBJ) and the Protein Data Bank (DDBJ: LC641776, BR001757, BR001749, and BR001750; Protein Data Bank: https://dx.doi.org/10.2210/pdb7dhw/pdb). All other study data are included in the article and/or supporting information.

## References

[1] M. Kollmar, S. Mühlhausen, Myosin repertoire expansion coincides with eukaryotic diversification in the Mesoproterozoic era. BMC Evol. Biol. 17, 211 (2017). 28870165 10.1186/s12862-017-1056-2 PMC5583752

[2] M. A. Hartman, J. A. Spudich, The myosin superfamily at a glance. J. Cell Sci. 125, 1627–1632 (2012). 22566666 10.1242/jcs.094300 PMC3346823

[3] J. Walklate, Z. Ujfalusi, M. A. Geeves, Myosin isoforms and the mechanochemical cross-bridge cycle. J. Exp. Biol. 219, 168–174 (2016). 26792327 10.1242/jeb.124594 PMC6514470

[4] T. Haraguchi , Molecular characterization and subcellular localization of *Arabidopsis* class VIII myosin, ATM1. J. Biol. Chem. 289, 12343–12355 (2014). 24637024 10.1074/jbc.M113.521716 PMC4007431

[5] L. Golomb, M. Abu-Abied, E. Belausov, E. Sadot, Different subcellular localizations and functions of Arabidopsis myosin VIII. BMC Plant Biol. 8, 3 (2008). 18179725 10.1186/1471-2229-8-3 PMC2275265

[6] D. Van Damme, F. Y. Bouget, K. Van Poucke, D. Inzé, D. Geelen, Molecular dissection of plant cytokinesis and phragmoplast structure: A survey of GFP-tagged proteins. Plant J. 40, 386–398 (2004). 15469496 10.1111/j.1365-313X.2004.02222.x

[7] A. Sattarzadeh, R. Franzen, E. Schmelzer, The Arabidopsis class VIII myosin ATM2 is involved in endocytosis. Cell Motil. Cytoskeleton 65, 457–468 (2008). 18393384 10.1002/cm.20271

[8] T. Shimmen, E. Yokota, Cytoplasmic streaming in plants. Curr. Opin. Cell Biol. 16, 68–72 (2004). 15037307 10.1016/j.ceb.2003.11.009

[9] D. Avisar, M. Abu-Abied, E. Belausov, E. Sadot, Myosin XIK is a major player in cytoplasm dynamics and is regulated by two amino acids in its tail. J. Exp. Bot. 63, 241–249 (2012). 21914656 10.1093/jxb/err265 PMC3245463

[10] M. Tominaga , Cytoplasmic streaming velocity as a plant size determinant. Dev. Cell 27, 345–352 (2013). 24229646 10.1016/j.devcel.2013.10.005

[11] V. V. Peremyslov, R. A. Cole, J. E. Fowler, V. V. Dolja, Myosin-powered membrane compartment drives cytoplasmic streaming, cell expansion and plant development. PLoS One 10, e0139331 (2015). 26426395 10.1371/journal.pone.0139331 PMC4591342

[12] J. Verchot-Lubicz, R. E. Goldstein, Cytoplasmic streaming enables the distribution of molecules and vesicles in large plant cells. Protoplasma 240, 99–107 (2010). 19937356 10.1007/s00709-009-0088-x

[13] K. Yamamoto , *Chara* myosin and the energy of cytoplasmic streaming. Plant Cell Physiol. 47, 1427–1431 (2006). 16963465 10.1093/pcp/pcl006

[14] N. Kamiya, K. Kuroda, Velocity distribution of the protoplasmic streaming in *Nitella* cells. Bot. Mag. Tokyo 69, 544–554 (1956).

[15] N. Kamiya, “Protoplasmic streaming” in Handbuch der Planzenphysiologie, F. Ruhland, Ed. (Springer, Berlin, 1962) pp. 979–1035.

[16] R. E. Williamson, Actin in the alga, *Chara corallina*. Nature 248, 801–802 (1974). 4835552 10.1038/248801a0

[17] K. Yamamoto, S. Hamada, T. Kashiyama, Myosins from plants. CMLS Cell. Mol. Life Sci. 56 227–232 (1999). 11212350 10.1007/s000180050424 PMC11146791

[18] S. Higashi-Fujime , The fastest actin-based motor protein from the green algae, *Chara*, and its distinct mode of interaction with actin. FEBS Lett. 375, 151–154 (1995). 7498467 10.1016/0014-5793(95)01208-v

[19] T. Q. Uyeda, Ultra-fast *chra* myosin: A test case for the swinging lever arm model for force production by myosin. J. Plant Res. 109, 231–239 (1996).

[20] T. Kashiyama, N. Kimura, T. Mimura, K. Yamamoto, Cloning and characterization of a myosin from characean alga, the fastest motor protein in the world. J. Biochem. 127, 1065–1070 (2000). 10833276 10.1093/oxfordjournals.jbchem.a022699

[21] M. Morimatsu , The molecular structure of the fastest myosin from green algae, *Chara*. Biochem. Biophys. Res. Commun. 270, 147–152 (2000). 10733919 10.1006/bbrc.2000.2391

[22] K. Ito , Recombinant motor domain constructs of *Chara corallina* myosin display fast motility and high ATPase activity. Biochem. Biophys. Res. Commun. 312, 958–964 (2003). 14651964 10.1016/j.bbrc.2003.10.202

[23] K. Ito , Kinetic mechanism of the fastest motor protein, *Chara* myosin. J. Biol. Chem. 282, 19534–19545 (2007). 17488711 10.1074/jbc.M611802200

[24] K. Ito, Y. Yamaguchi, K. Yanase, Y. Ichikawa, K. Yamamoto, Unique charge distribution in surface loops confers high velocity on the fast motor protein *Chara* myosin. Proc. Natl. Acad. Sci. U.S.A. 106, 21585–21590 (2009). 19955408 10.1073/pnas.0910787106 PMC2799862

[25] T. D. Schindler, L. Chen, P. Lebel, M. Nakamura, Z. Bryant, Engineering myosins for long-range transport on actin filaments. Nat. Nanotechnol. 9, 33–38 (2014). 24240432 10.1038/nnano.2013.229 PMC4914611

[26] P. V. Ruijgrok , Optical control of fast and processive engineered myosins in vitro and in living cells. Nat. Chem. Biol. 17, 540–548 10.1038/s41589-021-00740-7. (2021). 33603247 PMC10807509

[27] T. Nishiyama , The *Chara* genome: Secondary complexity and implications for plant terrestrialization. Cell 174, 448–464 (2018). 30007417 10.1016/j.cell.2018.06.033

[28] S. Kato , Morphology and molecular phylogeny of *Chara altaica* (*Charales, Charophyceae*), a monoecious species of the section Desvauxia. Cytologia (Tokyo) 75, 211–220 (2010).

[29] S. Kato , New distributional records, taxonomy, morphology, and genetic variations of the endangered brackish-water species *Lamprothamnium succinctum* (*Charales: Charophyceae*) in Japan. J. Asia-Pac. Biodivers. 14, 15–22 (2021).

[30] M. T. Casanova, K. G. Karol, A revision of Chara sect. Protochara, comb. et stat. nov (*Characeae: Charophyceae*). Australian Systematic Botany 27, 23–27 (2014).

[31] K. Yamamoto, M. Kikuyama, N. Sutoh-Yamamoto, E. Kamitsubo, Purification of actin based motor protein from *Chara corallina.* Proc. Jpn. Acad. 70, 175–180 (1994).

[32] A. Bateman , The Pfam protein families database. Nucleic Acids Res. 32, D138–D141 (2004). 14681378 10.1093/nar/gkh121 PMC308855

[33] M. Bähler, A. Rhoads, Calmodulin signaling via the IQ motif. FEBS Lett. 513, 107–113 (2002). 11911888 10.1016/s0014-5793(01)03239-2

[34] T. Haraguchi , Functional diversity of class XI myosins in *Arabidopsis thaliana*. Plant Cell Physiol. 59, 2268–2277 (2018). 30398666 10.1093/pcp/pcy147 PMC6217714

[35] T. Q. P. Uyeda, P. D. Abramson, J. A. Spudich, The neck region of the myosin motor domain acts as a lever arm to generate movement. Proc. Natl. Acad. Sci. U.S.A. 93, 4459–4464 (1996). 8633089 10.1073/pnas.93.9.4459 PMC39560

[36] K. C. Holmes, The swinging lever-arm hypothesis of muscle contraction. Curr. Biol. 7, R112–R118 (1997). 9081660 10.1016/s0960-9822(06)00051-0

[37] A. Houdusse , Crystal structure of apo-calmodulin bound to the first two IQ motifs of myosin V reveals essential recognition features. Proc. Natl. Acad. Sci. U.S.A. 103, 19326–19331 (2006). 17151196 10.1073/pnas.0609436103 PMC1687203

[38] Y. M. Kersey, P. K. Hepler, B. A. Palevitz, N. K. Wessells, Polarity of actin filaments in Characean algae. Proc. Natl. Acad. Sci. U.S.A. 73, 165–167 (1976). 1061112 10.1073/pnas.73.1.165 PMC335861

[39] J. R. Sellers, B. Kachar, Polarity and velocity of sliding filaments: Control of direction by actin and of speed by myosin. Science 249, 406–408 (1990). 2377894 10.1126/science.2377894

[40] T. Fujii, K. Namba, Structure of actomyosin rigour complex at 5.2 Å resolution and insights into the ATPase cycle mechanism. Nat. Commun. 8, 1–11 (2017). 28067235 10.1038/ncomms13969 PMC5227740

[41] P. S. Gurel , Cryo-EM structures reveal specialization at the myosin VI-actin interface and a mechanism of force sensitivity. eLife 6, e31125 (2017). 29199952 10.7554/eLife.31125 PMC5762158

[42] J. Robert-Paganin , The actomyosin interface contains an evolutionary conserved core and an ancillary interface involved in specificity. Nat. Commun. 12, 1–11 (2021). 33767187 10.1038/s41467-021-22093-4 PMC7994445

[43] S. Pospich, H. L. Sweeney, A. Houdusse, S. Raunser, High-resolution structures of the actomyosin-V complex in three nucleotide states provide insights into the force generation mechanism. *eLife* **10**, e73724 (2021). 10.7554/eLife.73724 PMC8735999 34812732

[44] T. Q. Uyeda, K. M. Ruppel, J. A. Spudich, Enzymatic activities correlate with chimaeric substitutions at the actin-binding face of myosin. Nature 368, 567–569 (1994). 8139694 10.1038/368567a0

[45] C. T. Murphy, J. A. Spudich, The sequence of the myosin 50-20K loop affects Myosin’s affinity for actin throughout the actin-myosin ATPase cycle and its maximum ATPase activity. Biochemistry 38, 3785–3792 (1999). 10090768 10.1021/bi9826815

[46] P. B. Joel, H. L. Sweeney, K. M. Trybus, Addition of lysines to the 50/20 kDa junction of myosin strengthens weak binding to actin without affecting the maximum ATPase activity. Biochemistry 42, 9160–9166 (2003). 12885250 10.1021/bi034415j

[47] C. M. Yengo, H. L. Sweeney, Functional role of loop 2 in myosin V. Biochemistry 43, 2605–2612 (2004). 14992598 10.1021/bi035510v

[48] K. Ajtai, S. P. Garamszegi, S. Watanabe, M. Ikebe, T. P. Burghardt, The myosin cardiac loop participates functionally in the actomyosin interaction. J. Biol. Chem. 279, 23415–23421 (2004). 15020589 10.1074/jbc.M310775200

[49] X. Liu, S. Shu, M. Kovács, E. D. Korn, Biological, biochemical, and kinetic effects of mutations of the cardiomyopathy loop of *Dictyostelium* myosin II: Importance of ALA400. J. Biol. Chem. 280, 26974–26983 (2005). 15897189 10.1074/jbc.M504453200 PMC1201472

[50] H. Onishi, S. V. Mikhailenko, M. F. Morales, Toward understanding actin activation of myosin ATPase: The role of myosin surface loops. Proc. Natl. Acad. Sci. U.S.A. 103, 6136–6141 (2006). 16603626 10.1073/pnas.0601595103 PMC1434513

[51] S. Struchholz , Functional role of the extended loop 2 in the myosin 9b head for binding F-actin. J. Biol. Chem. 284, 3663–3671 (2009). 19059909 10.1074/jbc.M808338200

[52] M. Tominaga , Higher plant myosin XI moves processively on actin with 35 nm steps at high velocity. EMBO J. 22, 1263–1272 (2003). 12628919 10.1093/emboj/cdg130 PMC151065

[53] S. Mühlhausen, M. Kollmar, Whole genome duplication events in plant evolution reconstructed and predicted using myosin motor proteins. BMC Evol. Biol. 13, 202 (2013). 24053117 10.1186/1471-2148-13-202 PMC3850447

[54] M. Kollmar, U. Dürrwang, W. Kliche, D. J. Manstein, F. J. Kull, Crystal structure of the motor domain of a class-I myosin. EMBO J. 21, 2517–2525 (2002). 12032065 10.1093/emboj/21.11.2517 PMC126035

[55] M. Levitt, Conformational preferences of amino acids in globular proteins. Biochemistry 17, 4277–4285 (1978). 708713 10.1021/bi00613a026

[56] K. Imai, S. Mitaku, Mechanisms of secondary structure breakers in soluble proteins. Biophysics (Nagoya-shi) 1, 55–65 (2005). 27857553 10.2142/biophysics.1.55 PMC5036629

[57] Z. Duan, K. Ito, M. Tominaga, Heterologous transformation of *Camelina sativa* with high-speed chimeric myosin XI-2 promotes plant growth and leads to increased seed yield. Plant Biotechnol (Tokyo) 37, 253–259 (2020). 33088188 10.5511/plantbiotechnology.20.0225b PMC7557661

[58] A. Lupas, M. Van Dyke, J. Stock, Predicting coiled coils from protein sequences. Science 252, 1162–1164 (1991). 2031185 10.1126/science.252.5009.1162

[59] K. Collins, J. R. Sellers, P. Matsudaira, Calmodulin dissociation regulates brush border myosin I (110-kD-calmodulin) mechanochemical activity in vitro. J. Cell Biol. 110, 1137–1147 (1990). 2139032 10.1083/jcb.110.4.1137 PMC2116058

[60] T. Lin, N. Tang, E. M. Ostap, Biochemical and motile properties of Myo1b splice isoforms. J. Biol. Chem. 280, 41562–41567 (2005). 16254000 10.1074/jbc.M508653200

[61] T. Q. Uyeda, S. J. Kron, J. A. Spudich, Myosin step size. Estimation from slow sliding movement of actin over low densities of heavy meromyosin. J. Mol. Biol. 214, 699–710 (1990). 2143785 10.1016/0022-2836(90)90287-V

[62] K. Ito, T. Q. Uyeda, Y. Suzuki, K. Sutoh, K. Yamamoto, Requirement of domain-domain interaction for conformational change and functional ATP hydrolysis in myosin. J. Biol. Chem. 278, 31049–31057 (2003). 12756255 10.1074/jbc.M304138200

[63] H. L. Sweeney , Kinetic tuning of myosin via a flexible loop adjacent to the nucleotide binding pocket. J. Biol. Chem. 273, 6262–6270 (1998). 9497352 10.1074/jbc.273.11.6262

[64] F. Wang, E. V. Harvey, M. A. Conti, D. Wei, J. R. Sellers, A conserved negatively charged amino acid modulates function in human nonmuscle myosin IIA. Biochemistry 39, 5555–5560 (2000). 10820029 10.1021/bi000133x

[65] E. Golomb , Identification and characterization of nonmuscle myosin II-C, a new member of the myosin II family. J. Biol. Chem. 279, 2800–2808 (2004). 14594953 10.1074/jbc.M309981200

[66] S. Komaba, A. Inoue, S. Maruta, H. Hosoya, M. Ikebe, Determination of human myosin III as a motor protein having a protein kinase activity. J. Biol. Chem. 278, 21352–21360 (2003). 12672820 10.1074/jbc.M300757200

[67] T. Sakamoto, I. Amitani, E. Yokota, T. Ando, Direct observation of processive movement by individual myosin V molecules. Biochem. Biophys. Res. Commun. 272, 586–590 (2000). 10833456 10.1006/bbrc.2000.2819

[68] S. Watanabe, K. Mabuchi, R. Ikebe, M. Ikebe, Mechanoenzymatic characterization of human myosin Vb. Biochemistry 45, 2729–2738 (2006). 16489766 10.1021/bi051682b

[69] R. S. Rock , Myosin VI is a processive motor with a large step size. Proc. Natl. Acad. Sci. U.S.A. 98, 13655–13659 (2001). 11707568 10.1073/pnas.191512398 PMC61096

[70] I. P. Udovichenko, D. Gibbs, D. S. Williams, Actin-based motor properties of native myosin VIIa. J. Cell Sci. 115, 445–450 (2002). 11839794 10.1242/jcs.115.2.445

[71] P. L. Post, G. M. Bokoch, M. S. Mooseker, Human myosin-IXb is a mechanochemically active motor and a GAP for rho. J. Cell Sci. 111, 941–950 (1998). 9490638 10.1242/jcs.111.7.941

[72] K. Homma, J. Saito, R. Ikebe, M. Ikebe, Motor function and regulation of myosin X. J. Biol. Chem. 276, 34348–34354 (2001). 11457842 10.1074/jbc.M104785200

[73] A. Herm_Gotz , *Toxoplasma gondii* myosin A and its light chain: A fast, single-headed, plus-end-directed motor. Embo J. 21, 2149–2158 (2002). 11980712 10.1093/emboj/21.9.2149 PMC125985

