# Supplementary material for: Discovery of ultrafast myosin, its amino acid sequence, and structural features
Source: Proc Natl Acad Sci U S A. 2022 Feb 16;119(8):e2120962119. doi: 10.1073/pnas.2120962119 (PMC8872768; doi:10.1073/pnas.2120962119)
Supplement: Supplementary File [file pnas.2120962119.sapp.pdf]

PNAS
www.pnas.org

Supplementary Information for
Discovery of the fastest myosin, its amino acid sequence, and structural features

Takeshi Haraguchi, Masanori Tamanaha, Kano Suzuki, Kohei Yoshimura, Takuma Imi, Motoki Tominaga, Hidetoshi Sakayama, Tomoaki Nishiyama, Takeshi Murata, and Kohji Ito

Corresponding authors:
Kohji Ito, E-mail: k-ito@faculty.chiba-u.jp
Takeshi Murata, E-mail: t.murata@faculty.chiba-u.jp

This PDF file includes:

Materials and Methods
Supplementary Text
Figures S1 to S5
Tables S1 to S4
Movies S1
S1 References

Table of contents

Materials and Methods 3
RNA extraction.. 3
Cloning of motor domain of *Chara braunii* myosins... 3
Protein engineering, Expression, and Purification.. 3
Crystallization and Data Collection... 5
The phylogenetic tree of *Chara* myosin XIs and the whole of myosin XIs............ 6
Identification of full-length sequence of *Cb*XI-4.. 7
Supplementary Text 8
Amino acids sequence of *Cb*XI-1.. 8
Amino acids sequence of *Cb*XI-2.. 9
Amino acids sequence of *Cb*XI-3.. 11
Amino acids sequence of *Cb*XI-4.. 12
Amino acids sequence of *Cc*XI.. 14
Figure S1. Schematic diagrams showing native *Cb*XI and recombinant *Cb*XIs. 16
Figure S2. SDS-PAGE of purified myosins. 17
Figure S3. SDS-PAGE of purified chimeric *Cb*XI-1showing calmodulin binding. ….18
Figure S4. Interaction of CM loop and loop4 of various myosins with actin.……….. 19
Figure S5. Structural comparison of pre-power stroke state and rigor state…………. 20
Table S1. Analysis of the IQ motif sequences of myosins using Pfam database……. 21
Table S2. Crystallographic data collection and refinement statistics for *At* XI-2 MD.. 22
Table S3. List of amino acids interacting between CM loop and loop 4 of myosins and actin……………………………………………………………………………. 23
Table S4. Expression levels of *Cb*XIs in *Chara braunii*... 25
Movies S1. *in vitro* motility assay of chimeric *Cb*XI-1……………………………….. 26
References for Supplementary Information.. 27

Materials and Methods

RNA extraction

Thalli of strain S276 were harvested in soil-water medium for the Charales (SWC-3) (1) under controlled laboratory conditions at 23ºC with a 16-h light: 8-h dark cycle with 24.5 μmol photons m^{-2} s^{-1} illumination provided by fluorescent lamps. The upper parts of thalli with reproductive organs were collected, frozen in liquid nitrogen, and stored at -80ºC until further processing. Frozen samples were ground in liquid nitrogen. Total RNAs were then extracted with ISOGEN (Nippon Gene, Tokyo, Japan), and purified using the QIAGEN RNeasy Plant Mini Kit.

Cloning of motor domain of *Chara braunii* myosins

cDNA of *Cb*XI-1 (g50407), *Cb*XI-2 (g48390), *Cb*XI-3 (g24025), and *Cb*XI-4 (g48658) motor domains (MDs) were amplified from total RNA of *Chara braunii*. The primers sequences used are as follows: 5'-TAGCGTCTCTTCAAAATGGCA-3' and 5'-CGCACTCTCCTTGTCATCTTCTT-3' for *Cb*XI-1, 5'-TATTTATAGTTCAGAATGGCGGAGC-3' and 5'-CCTGCGGCCAATTCTTTT-3' for *Cb*XI-2, 5'-CTCAGGAGTGTCACCATGGG-3' and 5'-ACTCTGCGCTGGATCTTGAC-3' for *Cb*XI-3, 5'-CTCACTCAGAATCATCATGGGGTC-3' and 5'-CTTCATTCTCTCATAGTCTTTACGCATCAG-3' for *Cb*XI-4.

Protein engineering, Expression, and Purification

*Cb*XI-1, *Cb*XI-2, *Cb*XI-3, *Cb*XI-4 MDs and chimeric *Cb*XI-1

A baculovirus transfer vector for *Cb*XI-1, *Cb*XI-2, *Cb*XI-3, *Cb*XI-4 MDs (Fig. S1B) were generated as follows: The cDNAs of motor domains of *Cb*XI-1 (amino acid residues 1–745 of *Cb*XI-1), *Cb*XI-2 (amino acid residues 1–751 of *Cb*XI-2), *Cb*XI-3 (amino acid residues 1–747 of *Cb*XI-3), *Cb*XI-4 (amino acid residues 1–742 of *Cb*XI-4) with optimized insect *Trichoplusia ni* codons were artificially synthesized by Eurofins Genomics because the expression levels in insect cells were very low when native cDNAs were used. These were cloned into the pFastBac MD (2) using the In-fusion cloning kit (Takara). The

resulting constructs, pFastBac *Cb*XI-1, *Cb*XI-2, *Cb*XI-3, *Cb*XI-4 MDs encode an N-terminal amino acids (MDYKDDDDKRS) containing the FLAG tag (DYKDDDDK), amino acid residues 1–745, 1–751, 1–747 or 1–742 of *Cb*XI-1, *Cb*XI-2, *Cb*XI-3, *Cb*XI-4, respectively, and C-terminal amino acids (GGGEQKLISEEDLHHHHHHHHSRMDEKTTGWRGGHVVEGLAGELEQLRARLEHHPQGQREPSR) containing a flexible linker (GGG), a Myc-epitope sequence (EQKLISEEDL), His tag (HHHHHHHH) and SBP tag (MDEKTTGWRGGHVVEGLAGELEQLRARLEHHPQGQREP).

A baculovirus transfer vector for chimeric *Cb*XI-1 (Fig. S1B) was generated as follows: cDNA of *Cb*XI-1 was mutated to create annealing sites at the 3' end of the MD of *At*XI-F and the 5' end of the C-terminal of coiled-coil of *At*XI-F. This was ligated to pFastBac *At*XI-F-HMM (3) using the In-Fusion cloning kit (Takara). The resulting construct pFastBac chimeric *Cb*XI-1 encodes N-terminal amino acids (MDYKDDDDKRS) containing the FLAG tag, amino acid residues 1–741 of *Cb*XI-1, amino acid residues 735–1,093 of *At*XI-F(UNIPROT ID: F4IRU3)., and C-terminal amino acids (GGGEQKLISEEDLHHHHHHHHSRMDEKTTGWRGGHVVEGLAGELEQLRARLEHHPQGQREPSR) containing a flexible linker, a Myc-epitope sequence, His tag and SBP tag.

A baculovirus transfer vector for *Cb*XI-1 6IQ was generated as follows: The cDNAs of 6IQ motif of *Cb*XI-1 (amino acid residues 746–879 of *Cb*XI-1) with optimized insect *Trichoplusia ni* codons were artificially synthesized by Eurofins. This was cloned into the pFastBac Flag *Cb*XI-1 MD Myc His SBP (this paper) using the In-fusion cloning kit (Takara). The resulting construct pFastBac *Cb*XI-1 6IQ encode N-terminal amino acids (MDYKDDDDKRS) containing the FLAG tag, amino acid residues 1–879 of *Cb*XI-1, C-terminal amino acids (GGGEQKLISEEDLHHHHHHHHSRMDEKTTGWRGGHVVEGLAGELEQLRARLEHHPQGQREPSR) containing a flexible linker, a Myc-epitope sequence, His tag and SBP tag.

<u>*Cc*XI MD with loop mutants</u>

*Cc*XI MD with loop 2 of *Cb*XI-1, *Cc*XI MD with loop 3 of *Cb*XI-1, *Cc*XI MD with loop 4 of *Cb*XI-1, *Cc*XI MD with loop 4 of *Cb*XI-1, and *Cc*XI MD with loop 4 and CM loop of *Cb*XI-1 were synthesized by Eurofins Genomics. These were cloned into pFastBac *Cc*XI MD (4) using the In-Fusion cloning kit (Takara).

<u>*At*XI-2 MD</u>

A baculovirus vector for *At*XI-2 MD motor domain (pFastBac Flag His TEV *At*XI-2 MD) was generated as follows: cDNA of *At*XI-2 (AGI code: AT5g43900.1, UNIPROT ID: Q9LKB9) was cloned from Arabidopsis seedlings. The motor domain of *At*XI-2 myosin contains residues 1-738 of the *At*XI-2 myosin heavy chain. The motor domain of *At*XI-2 was amplified by PCR. The gel-purified PCR fragment was cloned into the pFastBac vector using the In-fusion cloning kit (Takara). The resulting protein, *At*XI-2 MD has N-terminal amino acids (MDYKDDDDKRSMHHHHHHDYDIPTTENLYFQGA) containing the FLAG tag, His tag and TEV (ENLYFQG), amino acid residues 1-738 of *At*XI-2. *At*XI-2 MD was expressed in insect cells (High Five^{TM}, Life Technologies) and purified using nickel-affinity and FLAG-affinity resins as previously described (4).

These constructs were expressed in insect cells (High Five^{TM}, Life Technologies) and purified using Ni-affinity and FLAG-affinity resins as previously described (2, 4, 5). *Cb*XI-1 6IQ and chimeric *Cb*XI-1 were co-expressed with *Arabidopsis* calmodulin, CaM3 (AT3G56800) was as shown previously (5).

Crystallization and Data Collection

The purified sample was incubated with 200 µM ADP, 1 mM $AlCl_3$, and 5 mM NaF in this order for every 30 min on ice. The protein solution (0.1 µl; 4 mg/ml) was mixed with the reservoir solution (0.1 µl) consisting of 0.1 M MMT buffer (pH 6.0; Molecular Dimensions) and 25% PEG-1500. Crystals had grown at 296 K in sitting drops by vapor diffusion. The crystals were soaked for 1 min in 0.1 M MMT buffer (pH 6.0), 25% PEG-1500, 200 µM ADP, 1 mM $AlCl_3$, and 5 mM NaF and 20% glycerol, and were frozen and stored in liquid nitrogen.

The X-ray diffraction data were collected from a single crystal at a cryogenic temperature (100 K) on BL-17A at the Photon Factory (Tsukuba, Japan). The collected data were processed using XDS software (6). The structure was solved by molecular replacement with Phaser (7) as a search model for *Dictyostelium discoideum* MD complexes with ADP-AlF_4 (PDB ID code; 1MND). The atomic model was built using Coot (8) and iteratively refined using Phenix (9). TLS (Translation/Libration/Screw) refinement was performed in late stages of refinement. The refined structures were validated with RAMPAGE (10). Homology models were built using Modeller 9.18 (11). All molecular graphics were prepared using PyMOL (The PyMOL Molecular Graphics System, Version 2.1.1, Schrodinger, LLC, New York, NY, USA).

The phylogenetic tree of *Chara* myosin XIs and the whole of myosin XIs

Myosin homologs were collected from NCBI RefSeq database for *Arabidopsis thaliana*, *Amborella trichopoda*, and *Selaginella moellendorffii*, and *Physcomitrium patens* based on BLASTP search using the *Cb*XI-1 as a query. Another search for NCBI nr dataset were performed with organisms specified as Viridiplantae but excluding Spermatophyta. Further, ferns (*Salvinia cucullata* and *Azolla filiculoides*) (12), bryophytes (*Marchantia polymorpha* (13) and *Anthoceros agrestis* (14)) , and Zignematophycean algae (*Spirogloea muscicola* and *Mesotaenium endlicherianum*) (15) were searched for respective datasets. Those datasets were obtained from ftp://ftp.fernbase.org/Salvinia_cucullata/Salvinia_asm_v1.2, ftp://ftp.fernbase.org/Azolla_filiculoides/Azolla_asm_v1.1, https://phytozome.jgi.doe.gov/pz/portal.html#!info?alias=Org_Mpolymorpha, https://www.hornworts.uzh.ch/static/download/a_agr_oxford.zip, and https://figshare.com/articles/Genomes_of_subaerial_Zygnematophyceae_provide_insights_into_land_plant_evolution/9911876/1, respectively.

Thus, 80 sequences were retrieved and aligned with einsi in MAFFT v7.475 (16). The alignment was read with Mesquite version 3.61 (17) and well-aligned regions were marked as included after excluding all characters, to ensure only well-aligned regions were used for the subsequent analyses. Two sets of the matrix were extracted from the entire

alignment; one including myosin VIII, and another confined to myosin XI excluding sequences having deletions in conserved regions, which may be either alternative splicing or misannotation. Sequences identical in the included region were regarded as a single OTU and one representative was retained during the analysis. Thus, green plant myosin dataset included 567 sites from 43 OTU and myosin XI dataset included 1060 sites from 32 OTU. Substitution model was chosen using ProteinModelSelection.pl from RAxML distribution. Standard RAxML version 8.2.12 (18) was used for the maximum likelihood tree search with -f a -# 100 option and 24 bits of random seed were provided from system random source /dev/urandom to -p and -x options. 1000 bootstrap datasets were prepared using seqboot from PHYLIP version 3.697 (19) and split to individual dataset and then run essentially the same RAxML command. The bootstrap trees were collected along with the original tree amplified 1000 times to force the consensus topology and the bootstrap value was counted using consense then subtracted for 1000 to recover the bootstrap value.

Identification of full-length sequence of *Cb*XI-4

Initially, g48658 was identified as a Myosin XI homolog and designated *Cb*XI-4. The g48658 spanned almost entire range of the scaffold_743 and appeared to contain only N terminal part. Thus, the transcript sequence (2554 bp) was used as a query to search several transcriptome assemblies of *Chara braunii*, which were constructed during the genome project but was not described (ref genome paper). A BLASTN (20) search to an assembly constructed with TransAbyss (21) found a 4242-bp contig, which is considerably longer than the query. This sequence was used to search the predicted transcriptome and found that g66754 has partially matching sequence without scattered mismatches. g66754 was located on the minus strand at the 3’ end of scaffold_8 and RNA-seq data suggested transcripts spanned to g66753 and g66745. The prediction by augustus (22) contained exons that were not linked with RNA-seq data overlapping introns and having similarity to retrotransposons. Thus, the g66745-g66753-g66754 locus was manually edited on WebApollo (23) to conform with the RNA-seq data with intron support, and two isoforms were retained. This sequence was connected to the 4242-bp contig of the RNA-seq assembly to end up in the two full-length nucleotide sequence of the transcripts.

Supplementary Text

Amino acids sequence of *Cb*XI-1

>*Cb*XI-1, Full-length: 1 -2690, MD: 1 -745, 1 IQ: 746 - 761, 2 IQ: 766 - 784, 3 IQ: 790 - 810, 4 IQ: 814 - 825, 5 IQ: 838 - 858, 6 IQ: 862 - 877, Coiled-coil: 883 - 924, 929 - 975, 988 - 1079, 1161 - 1188, 1265 - 1292, GTD: 2322 - 2690

MATFGVGSPVWVEDEEDMWIEATVLKIEADKVISKNRKGGEVVSSKDMVHPRD
EDTAEMGVDDMTRLSYLHEPGVLDNLSRRYHLNQIYTYTGSICIAINPFQAVPHL
VGTKLMEMFKVAQPGEVSQPHVYAVADRAYKAMMDEEKSQSILVSGESGAGK
TEATKLIMNYLAFMGGRATPVAGERSVEQKVLESNPLLEAFGNAKTVRNNNSSR
FGKFVEIQFNRGKISGAAVRTYLLERSRITQVSTPERSYHCFYQLCAGATAEEREK
LKIEAAPNYFYLNQSECFEVPRFDEVEEYKATRHAMDVVGISTEEQDGIFRIVASI
LHLGNVDFKPGKEADSSQLADDKSRFHLNCCAELLGANPKLLEDSLIQRIMVTR
GEAITKLLDKKQAVGSRDALAKTLYAKMFDWLVDKVNKSIGQDPNSNTLVGVL
DIYGFESFTVNSFEQLCINLTNEKLQQHFNTHVFKMEQEEYVKEEINWDNIDFVD
NIDVLDLIEKKPLGIIALLDEACMLPKSTPESFGQKLAQSFDKHKRFTKHKFKKTL
FKIDHFAGEVEYSTDTFIEKNKDFVIAEHQQLLTASTDPFVRQVYPPPEEPKQGGK
GGGKSSFSSIGTRFKQQLQSLMDTLNQTEPHYVRCVKPNQKLKPLMFEKRIVLQ
QLRCSGVLEAVRISCAGFPTRRTFFEFADRFKILFPDAVANCGQDYKSACVKILE
KIGLERYQIGKTKVFLRAGQMAILDTKRTEILNEAAKKMTRRVRTFLARNEYLR
VKQASVCVQTRWRGVLAFKLFVFLKETRAATEMQRYVRGMQNFKAYREMKA
ASIRIQAAVRSLCAKNLYKQLQEHKAASIIQANWRACFATKSYKELLSSTILFQSA
WRSKNARADLFDLKELAKEIDVLQDTKQKLESRVADLTSELATETKTRMDLEAQ
RASEVAALEAQIADMRGQAEDTNKSIEKARRVHQENLDQIKRDADDELERGLAA
ELPAEAKKTIESLKQLNDKFLKVVKAATARTEEAEEKISAAEKKAAEALKEMQD
VNAAAKALREQKAKEDAAALATMQKEKQKQEEVTKRLETESNSLRKTADKMA
GLETDVVKVREETAAAEAVDTTEIEELRRTVADAVEIDPKEIEKLRKDSETVASL
ESEAEKLQEEVKNAPKVDPVELEELRAKVKKMEEDAAAAAAAAAAEAYQAEM
DSLKEPAKKATDLQAEVEKVKKELEAMAAPSLDASELETIKKEAEKLASTAAST
ACDPAELEKLRQEAAKVPALEAEAAGLQAQSQRVTVLEAENKSLQEQLSAAGV
TPAPAVAAAPAYPGATPPAAASAYPGQAAIANPPSYPGAPAAAAPPPYPGAPAA

AAPPAYPGAPAPVAAPVYPGAPATAAAPAYPGAPAPAPAPAYPGAPAPAPAPAY
PGAPAPAATPAYPGAPAPAASPAYPGAPAPAAAQAYPGAPAPAAAQAYPGAPAP
AATPAYPGGPAAVAAAPYPCPPPSPSPATYHGGQVAESYAAQSLFNATQPASMT
GTAPSPYLSTPEQQQQQQQVMMAAGPHAQQQMMASQQQQQQQQQQQQASFP
GARPGTPAQGVYPPQQQQGMMQQRAMTPDGKMGMQQQLGQQMASPQIMPQQ
QQQQAMMKMGGQSLFPQMGMGQQQSNGMSQDMQNVNGGAMGSQAGMGQQ
QTNFGVGGIGSMGMGMGMGMGNNMMTDAQGPGGQQGSADSQMAGALGGQ
MVNGPMNPLMQALSSQMGGMGNPLAQLAAQFGLGNATGPMGQMAPMGQAN
PLAQLLMGMTGMGGMGGNMGMQGMQSMGNMGMGMSGMGGHPGMDRGMD
RGMDRGMDRGMDRQSTSMKNPFGFASLAHDWASRARSNLHREEPMVFQISRE
DMDARSPELGYSMQDDDQMQLRSSQEGGFGRRDSTEGRRDSESMRDMERDYD
MRPGRDSDLRSSRDKEFESRRDSAYDLRGSRDYESREYEMRGSRELDMRRADSQ
RRGEYDSTRRESTGSRDFDPRRESREFDTRRSSLGSTREFDSRRDYDLRRESKDFD
PRRESREFDSRRHSGSRDFDGSRREFDTRRDFREFNDEPESRRDSRESDLRRDSLR
GSDLRRDSLRESESRREYRDLDSPTGRDLGARRERDLYSSRKESSRARSSQRDAD
DLGENEPRRSRRRSHNEPVHDSDGSPPPTKTRSSTRRSSRGDALTKADAVSRRES
VSELRSSRRDSTADPRASTTDLRGSRTSEARSSQKESRFREAVSELRSSRKENPKN
ERGPREDESMETENTGRDIPVVEGLEADAPADPDRKLSRSRSVSSRQLLEQKRRK
AALEKLQKDQGLLIHCISQDVGFSLNFPVAAGVVFKSLLHWNSFKSEHTRIFSRV
VAVVISGIEGALDNNDGLAYWLSNSTCLLNMMQRTLRFSSDRRRSSKSGIYGSG
TGVDTSNLMEAQRPALVFRQQLVACTEKIFGTIRDNLTKIVAPLLPVCLKVQKSR
RRSRVQAEDEVKVSNGYAAIVSTLDDLLDVMRKNMVFPVLTQSVVEQVFAFIN
VHMFNSLQMRRECCCYSNGLFVRDGLKMLDHWASDVGRQSSGRASEKLTHIRQ
ATHFLIYDDKETATLEDITKIVCPDLNMHQLHRIATMYSDDANVCPGLSPEVVDD
LADATQDSLTETFLLTDDTSTPFTLDDIVIATSASLGEMMSVEPPQLLADSPSFQF
LLSEAA

Amino acids sequence of *Cb*XI-2

>*Cb*XI-2, Full-length: 1 -2239, MD: 1 -751, 1 IQ: 752 - 767, 2 IQ: 772 - 786, 3 IQ: 797 - 811, 4 IQ: 820 - 831, 5 IQ: 844 - 864, 6 IQ: 869 - 885, Coiled-coil: 889 - 930, 994 -1021, 1051 - 1102, 1167 - 1215, 1635 - 1664, GTD: 1861 - 2239

MAEQVAIGSPVWVEDAEDMWIEAVVMKIEAKKITSKNIKGTIVESSPEMVHPRD
EDTKPLGVEDMTRLSYLHEPGVLDNLLRRYMEKEIYTYTGNICIAINPFQAVPHL
VGTQLMEIFKNSQPGEVTQPHVYAVADRAYKAMLDEGQSQSILVSGESGAGKT
EATKLIMNYLAFMGGRSGGAASSGGVRTVEQKVLESNPLLEAFGNAKTVRNNN
SSRFGKFVEIQFNGTKISGAAVRTYLLERSRVTQVSTPERSYHCFYQLCAGASEE
DRAKMKIGKASDYHYLNQSECFEVPRIDDKEEYSITRNAMDVVGISEEEQDGIFR
IVAAILHLGNIEFAPGKDADSSKIADEKSRYHLEACAELLACDPNLLEYSLIQRVM
MTGTEKIKKLLNKTAALGSRDALAKTLYAKMFDWLVQKVNVSIGQDATSTTLV
GVLDIYGFESFKVNSFEQLCINLTNEKLQQHFNNHVFKMEQQEYIREEINWSNID
FVDNIDVLDLIEKKPVGIIALLDEACMLPKSTPESFAAKLYGSFDKHKRFGKHKF
KKTLFRIDHFAGEVEYSTETFIEKNKDFVITEHQELLMNSKDPFIRDVYPPPPEEPK
QGGGKGGSKSSFSSIGTRFKQQLQSLMETLNATEPHYVRCVKPNQLLKPLNFDK
RIVLQQLRCSGVMEAVRISCAGFPTRRTFFEFQDRFKVLYPDVVAQCGEDYRLA
CVKVLERTGLEDYQIGKTKVFLRAGQMATLDARRQTILNAAAKRIGRRVRTFLA
REEYLRWRAGATVMQTRWRGVMAFNLFTWLRQMAAATEMQRYVRGMQAFG
RYREMKAASIRVQAAVRSLCAKNELHRLQEHRAASIMQANWRACFASKCYKEV
LSSSVVFQSAWRSKNARADLRDLKELASEIAVLTDVKQKLEGDLSGLQQELTKE
VGTRTDLEAQRAAEVTSLESTTEEVCKDVSATKAEIGKGREKHAEDLREIKQRSE
EDLEVGLSAELPPQAKKTIEGLKQTNEQFKKASEAATARIAAAMAKVTAAETAT
NECLNEMTEVNAAAAALRAQKAKEDAEAIAEMKAEIAKLEVDSKRLRSENTSL
ARVARRLDHLETQVEKFRDEEYEASNVDTTEIDELRRTVADVVEIDPKEIESLRK
KADSVAALEGEEEKLQDDIRKAPKIDPEEVERLKQQVQKWEEEAAAAAAVDA
AKRAAEAKTAELESLRAEVTRMNGIQADLVKMKSTLVSISVRQCLDAAEMEAIK
EEAKRSVVIPPPPEPPVVPLAEILALKTDAQKVPALEIELQSLKAQSQKVLTLEAE
NSTLREQAARAAEPPPPPQPSAAAPSPYGAAPQGSPVAVMAASGHQQHMTPPGS
PPMDAMRVSAEGPLSAQGTATVSQMRAGAVGMHPQSSMASNPMTPMMANPM
MANAMANPMMANPMANPMANPMMANQMMANPMMANPMMANPMMANPM

MANPMMANPMMAMQTNPMAQLMAMQMGMLNPMAAMMGFNPFMNPMMEQ
MGMSMGPMGHMGMMNPMAAMMMSQMGGGMDIPGMGPAAQSMAGSTADKS
NSNIKKKNAATSTGKKSSSKKRVAAADGSTGTTKSGRNAAAASTNGLGMGGM
GFGGMANFFNPMARMQEMFKMMQAQSSNASGSQNRDKDEEGNAEQQQQQQQ
EEEEEEDEEEEQLSLPNAMGADKLRMSRESRLHSKGRTSRGKSGNAALYRESVA
DLRSLRREKGREGMMGEGRLVRGKKMTDATRVAARAQRHSIADLSFAHATKG
TPVHASERGSATSREALPQGNGGESTVEDDYKEAAAAENAEPNGDASAPLVPDT
SAAVEAPEPVTPLIRSRTDVRMSQDQKKRRAMMEQIQRDQGLLVQSIMQDVGFE
HGIPVAAAVVFRCLLHWHSFESEHTRVFSRIINVVVSGIQTTMDSNDGLGYWLSN
TSVLLVLMQQTLRFSSGRRSLSRKSMIYGSGKGHLGMEMAKNIEGQRPAMVFK
QQVNACLERAFGIIRDNVTKDIFGHLSACIKAPKKSPDNSPLRLSRNSLVTEDDGS
SGGWGSIIPILDQFMNSMKANFVLPVLVRKLFIQIFGFMDINLFNSIIMRRECCSYS
NGEFIMEGLMEIKLWLDDREQYAGGAWEELAHIRQAADFLVYKQKSSASADELI
ANLCSSLTIHQIHRLASLYVDNTNMRPGLSPEVLQTLADKSHSSFADSCMLADDE
RIPFSVDEIIDDAALSTREVMEVQAPTMLRENPSFRFLVDVTD

Amino acids sequence of *Cb*XI-3

>*Cb*XI-3, Full-length: 1 -1625, MD: 1 -747, 1 IQ: 748 - 764, 2 IQ: 768 - 787, 3 IQ: 794 - 811, 4 IQ: 815 - 831, 5 IQ: 842 - 859, 6 IQ: 864 - 882, Coiled-coil: 885 - 912, 920 - 972, 979 - 1110, GTD: 1230 - 1625

MGSEEVRAPGGASAVLAVGSPVWIEDPEKAWIEATVVKIDGVVVTARTINGDLV
ETTLANGIPRDEDVTMRGVDDMTKLSYLHEPGVLHNLYTRYKHDEIYTYTGNIL
IAVNPFTRLPHLFNQYMMKQYQDAQPGDLNPHVYSVAGAAYKAMMDENKSQS
ILVSGESGAGKTETTKQIMQYLAFVGGRTVGDNRSVERQVLQSNPLLEAFGNAK
TVRNNNSSRFGKFVEIQFNKGKISGAAVRTYLLERSRVTQISTPERNYHCFYQLV
AGASPEDVERLKLGPPESFHYLNQSKCVEVGTIDDCKEYQLTREAMDVVGIGAE
EQEAIFRTIAAVLHLGNIEFSTGASEASEVSSEKAKFHLRAAAEILMCDEKMLEKS
LTTRIMRASRTESITKILDKSQATDNRDALARTIYAKLFDWLVDKVNKSIGQDLH
STVLIGVLDIYGFESFDINSFEQFCINLTNEKLQQHFNTHVFKMEQAEYRKEEINW
DDIDFVDNIDVLDLIEKKPGGIIALLDEACMFPKSTAESFASKLGSTFQSHRRFSRP

KFKRTAFTIDHYAGQVEYRADLFLEKNKDYVVPEHQQLLHASKCPFVAALFPPD
EGTKAPSKFASIGSQFRLQLASLMDTLKLTAPHYIRCVKPNMQLKPQIFENKNVL
QQLRCSGVLEAVRISCAGFPTRRTFGDFLDRFGLLHPEVLVESADEKAACQILLE
KCNLKGYQIGKTKVFLRAGQMAILDTKRSNVLNEAAVKIQRRVKTFLMSRDYQ
RRKDASVLVQAYWRGTMARLEFRFLREQISAICFQRYIRGYLARKNYLEMRQAA
IRIQSAVRSLAARTVLCTLQDNFAATQIQSKWRSYLASRSYNGLLRSCIFFQGAW
RCKEARSELKKLRQAARETGALREAKNRLEKKCEELTLRLGLSRDDLIAKNNEL
AKLQSAMEEMQVQAEQMKALMAKEREVYEAELAQAKATAAQLVDAEMAAQP
SKEVLEEIKALTEENEKLKKHVEDYEKKKALAERSAKRIEEADSKLDTMQELLG
RMEEQVQNLISENQSLQSEKQNLQSENRILMQQALSMKDLESENQDLQRNLKHL
EADSQALKSENQALKQQLEQRTWTNNKDSSAAVKVGPSPLVSPRPIERIPAFSNG
LPQIKVITPAADPVPAEKIWMPKPKIVVSAPHADGQLREETPRTPAYAPPVASLGS
PGVKAPAVDHKISKMMHDKLQNDQEALLACVMQDVGFSNDHPVAAVIIFKCLL
QWHSFEAERTNVFDRIISAIQTAIDSHSGNNDVLGYWLSNTSTLLYLLQRTLKAS
GGGGTAPQRRRQTTLFGRMTQRFSSQQQNYPNGMGPIGLDNVRQVEAKYPALL
FKQQLTAYVEKIYGMVRDNLKKEITPLLGSCIQAPRAPRHPLGRKVSPAPGQQVL
SSHWGSIINSLLKLLNILRGNKVPPYLVRKIFTQIFSFINVQLFNSLLLRRECCSFSN
GEYIKAGLAQLEHWIYEAGEEYAGASWEELRYIRQAVGFLVIHQKPKKSLDEIM
HDLCPALSIQQLYRISTMYWDDKYGTHTVAPEVIQNMRILMTEDSNNAVSNSFL
LDDDSGIPFSVDDISKSMPEVDLSEVYPPPLLKDIPSFRFLQPEKE

Amino acids sequence of *Cb*XI-4

>*Cb*XI-4, Full-length: 1 -1815, MD: 1-742, 1 IQ: 743 - 758, 2 IQ: 764 - 782, 3 IQ: 789 - 806, 4 IQ: 811 - 827, 5 IQ: 836 - 854, 6 IQ: 859 - 875, Coiled-coil: 880 - 907, 915 - 949, 974 - 1105, GTD: 1420 - 1815

MGSEEARSAALGIGSPVWVEDVENAWIEATVVKRDGDAVTARTVHGDLVETR
MANALPRDEDVTMRGVDDMTKLSYLHEPGVLHNLYARYKHDEIYTYTGNILIA
VNPFTRLPHLFNTYMMKQYQDAQPGDLNPHVYSVADAAYKAMMEEMKSQSIL
VSGESGAGKTETTKQIMQYLAFVGGRTVGDNRSVEQQVLQSNPLLEAFGNAKT
VRNNNSSRFGKFVEIQFNNGKISGAAVRTYLLERSRVTQISTPERNYHCFYQLVA

GASHEDAERLKLGPPDSFHYLNQSKCVEVGAIDDCKEYQLTREAMDIVGITTEE
QEAIFRTIAAVLHLGNIEFNSGESEASEVSTEKSKFHLKAAADMLMCDEEMLEKS
LTTRIMKATRTESITKILNKSQATDNRDALARTIYAKLFDWLVNKVNKSIGQDPH
STVLIGVLDIYGFESFEINSFEQFCINLTNEKLQQHFNTHVFKMEQAEYRKEEINW
DDIDFVDNIDVLDLIEKRPLGIIALLDEACMLPRSTAESFARKLGDTFHNHKRFSK
HKFKRTAFTIDHYAGQVEYRADLFLEKNKDFVVPEHQQLLHASKCAFVSGLFPL
DEGAKAPSKFMSIGSQFKLQLATLMETLKLAAPHYIRCVKPNMQLKPQIFENKN
VLQQLRCSGVLEAVRISCAGFPTRRTFEEFLDRFGLLHPEVLIESADEKVACQNLL
EKCNLKGYQIGKTKVFLRAGQMAILDTKRSNVLNEAAVKIQHMVQSFLMRKDY
ERMKSASLLVQAYWRGTMARLEFRFLREQVSAVCFQRYIRGYLAQKNYLKMQ
QAAIRIQSAVRSLAARRFLCFLEDNHAATQIQSKWRSYVAFRSYDELLRSCIVFQ
GAWRCKEARSEIKKLRQAARETGALREAKNRLEKKCEELTLRLGLAKVSLIARN
SELAKLQSAMEEAKAQVEQMKMLVAKERKGHEADLAQAKVAAAQLLDAKMS
AQPSKEVLDKIEALSEENTKLKKLVEDYEKKKALAESSAKRIEEADLKRDAMQE
LLSRSEEQVQNLISENQSLQSEKENLQSENRILRQQALNMKDLESENQDLQRNLT
HLETNSQALRAENETLKQQLEQLTSKGGTAVKIGQPAVTPWLEATDTVYGMSG
HETGVPRRDFRQYAGRGQVEGNKIVPNALHSQEELGTEPSSVHRLGPSARNTVV
EAKPEDSMIVADSANGLGNSDAKASAGTRMQTRADYVGAQDYVTHTQMMQPF
ESSPSTDDRNAPSPASQPLHCVESASVSTLDRPDHRRAAQGSRVDLLAQGPRQYE
SRTGQRDTGHSVYGSRPEPRTALSTGLSRLSHRPETGTLIKPTPEPVITTSYPDEQP
TTPGVTGPGTPSRPLGRSQHIRRESSDYMSLYLPEDSPVPEAKPVDQKKSKMMPD
KLQSDQEALFDCLMQDVGFSKDHPVAAVIIFKCLLQWHSFEAERTNVFDRIINAI
QTAIESHSDNNDVLAYWLSNTSTLLHLLQRTLKAGGGGGTTPQRRRQTTLFGRM
TQRFSSQQQNYPNGTGPIGLDNVRQVEAKYPALLFKQQLTAYVEKIYGMLRDNL
KKEITPLLGSCIQAPRAPRHQLGRKVSLAPGQQVLSSHWGSIINSLLKLLNILRGN
KVPPYLVRKTFTQIFSFINVQLLNSLLLRRECCSFSNGEYIKAGLAQLEHWIYEAG
EEYAGASWEELRYIRQAVGFLVIHQKPKKSLDEIMHDLCPALSIQQLYRISTMYW
DDKYGTHTVAPEVIQNMRILMTEDSNNAVSNSFLLDDDSGIPFSVDDISKSMPEV
DLSEVHPPPLLKGMPSFRFLQPEKA

Amino acids sequence of *Cc*XI

>*Cc*XI, Full-length: 1 -2182, MD: 1 -746, 1 IQ: 747 - 762, 2 IQ: 767 - 786, 3 IQ: 793 - 810, 4 IQ: 814 - 832, 5 IQ: 840 - 858, 6 IQ: 863 - 879, Coiled-coil: 884 - 911, 978 - 1208, 1234 - 1327, 1353 - 1446, 1472 - 1631, GTD: 1787 - 2182

MGLEKARSSALGIGSPAWVEDVETVWIEATVVKLDGDAITARTVNGDLVETTM
ANALPRDEDVTMRGVDDMTKLSYLHEPGVLHNLYTRFKHDEIYTFTGNILIAVN
PFTRLPHLFNTYMMKQYQDAQPGDLNPHVYSVADAAYKAMMEEMKSQAILVS
GESGAGKTETTKQIMQYLAFVGGRTVGDERSVEQQVLQSNPLLEAFGNAKTVR
NNNSSRFGKFVEIQFNNGKISGAAVRTYLLERSRVTQISSPERNYHCFYQLVAGA
SPEDAERLKLGPPDSFHYLNQSKCVEVGAIDDCKEYQLTREAMDIVGITTEEQEA
IFRTIAAVLHLGNIEFDSGESDASEVSTEKSKFHLKAAAEMLMCDEQMLEKSLTT
RIMKATRTESITKILNKSQATDNRDSIAKTIYAKLFDWLVNKVNKSIGQDPHSTV
LIGVLDIYGFESFEINSFEQFCINLTNEKLQQHFNTHVFKMEQAEYRKEEINWDNI
DFVDNIDVLDLIEKKPLGIIALLDEACMLPRSTAESFARKLGDTFNNHRRFSKHKF
KRTAFTIDHYAGQVEYRADLFLEKNKDFVVPEHQQLLHASRCAFVSGLFPADEG
TKAPSKFMSIGSQFKLQLAALMETLKLTAPHYIRCVKPNMQLKPQIFENKNVLQ
QLRCSGVLEAVRISCAGFPTRRTFEEFLDRFGLLHPEVLIESAEESADEKVACQNL
LEKCNLKGYQIGKTKVFLRAGQMAILDTLRSNVLNEAAVKIQHMVQSFLMRRD
YERMKRASLLVQAYWRGTMARMEFRFLREQVSAVCFQRYIRGYLAQKNYFEM
RQAAIRIQSAIRSLAARRVLCVLQDNHAATQIQSKWRSYVAFRSYDELLRSCKVF
QGAWRCKEARSEIKKLRQAARETGALREAKTRLEKKCEELTLRLGLAKVSLIAR
NSELAKLKFAMEGAQAQVEQMKILLAKEREGHEADLAQAKVAAAQLLEAEMS
AQASKEVLDKVEALSEENSKLKELVEDYEKKKALEESSAKRIEEADLKRDAIQEL
LNRSEEQVQDLISENQSLQSEKLNLQLDNRILRQQALSMKDLELEKQDLQRNLQ
HLEANSQALRAENQTLKQQLEQLESQDLQRNLQHLEANSQALRAENQTLKYQL
EQLESQDLQRDLQHLEANSQALRAENQTLKQQLEQLESQDLQRNLQHLEANSQ
ALRAENQTLKQQLEQLTSKGGTVLKIGGASSKFDISEPEPAIGETEFTEANSQALR
AENQTLKYQLEQLESQDLQRNLQHLEANSQALRAENQTLKQQLEQLESQDLQRT
LQHLEANSQALRAENQTLKQQLEQLTSKGGTVLKIGGASSKFDISEPEPAIGETEF
TEANSQALRAENQTLKQQLEQLESQDLQRNLQHLEANSQALRAENQTLKQQLE
QLESQDLQRNLQHLEANSHALRAENQTLKQQLEQLTSKGGTVLKIGGASSKFDIR
EPEPAIGETEFTEANSQALRAENQTLKQQLEQLESQDLQRNLQHLEANSQALRAE
NQTLKQQLEQLESQDLQRNLQHLEANSQALRAENQTLKQQLEQLESQDLQRNL
QHLEAKCQALRAENETLKQQLEQLESQDLQRNLQHLEANSQALRAENQTLKQQ
LEQLTSKGGTVVKIGRAAVTRIKPTPEPVITTSYPDEQPATPGVTGPGTPSRPLGRS
QHIRSESSDFTSLYFREDSPVPEAKPVDHEKSKMMPDKLQYLPEDSPVPEAKPVD
QKKSKMMPDKLQYLPEDSPVPEAKPVDQKKSKMMPDKLQSDQEALLDCLMQD
VGFSKDHPVAAVIIFKCLLQWHSFEAERTDVFDRIISAIQKAIESHSDNNDVLAYW
LSNTSTLLHLLQRTLKTGGGGGTTPRRRQATLFGRMTQRFSSQQENYPNGMGP
VGLDNVRQVEAKYPALLFKQQLSAYVEKIYGMLRDRLKKEITPLLGSCIQAPRAP
RHQLVRKLSLTPAQQVLSSHWGSIINSLLTLLNALRGNKVPPYLVRNIFTQIFSFIN
VQLVNSLLLRRECCSFSNGEYIKAGLAQLEHWIYEAGEEYAGDSWEELRYIRQA
VGFLVIHQKPKISLDEIINDLCPALSMQQLYRISTMYWDDKYGTHTVAPEVIQNM

RILMTEYSYNAGGNSFLLDDDSGIPFSVDDISKSMPDVDLSQVDPPPLLKNRPSFR
FLQPGKA

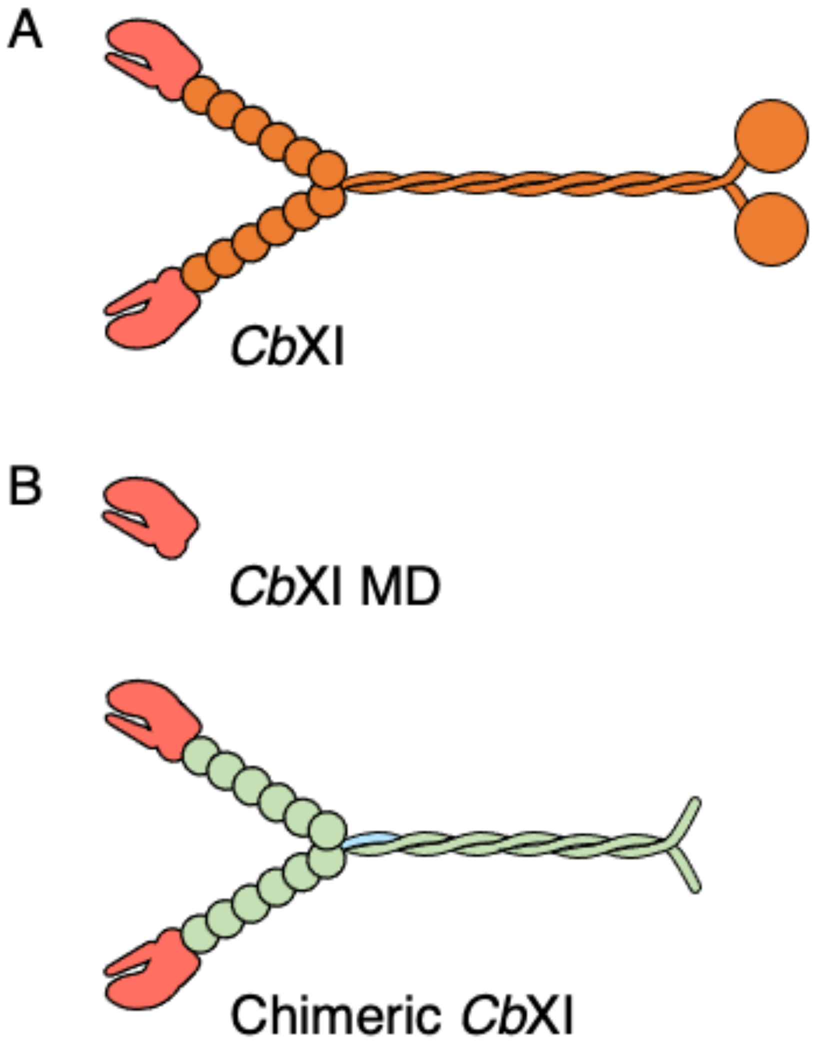

Figure S1. Schematic diagrams showing native *Cb*XI and recombinant *Cb*XI constructs. ***(A) Cb*XI**: the domain structure of the native *Cb*XIs deduced from its amino acid sequence as mentioned in Fig. 1. Each heavy chains of native *Cb*XIs have 6IQ motifs to which 6 light chains bind. The light chains that bind to each IQ motif are unknown. ***(B)*** recombinant *Cb*XI constructs. ***Cb*XI MD**: a construct containing only the MD of *Cb*XI. **Chimeric *Cb*XI**: a construct containing the MD of *Cb*XI (shown in red), and the 6IQ motifs and the coiled-coil domain of *At*XI-F (shown in green). It is thought that calmodulin bind to IQ motifs of the chimeric *Cb*XI because calmodulin binds to 6IQ motifs of *At*XI-F (3).

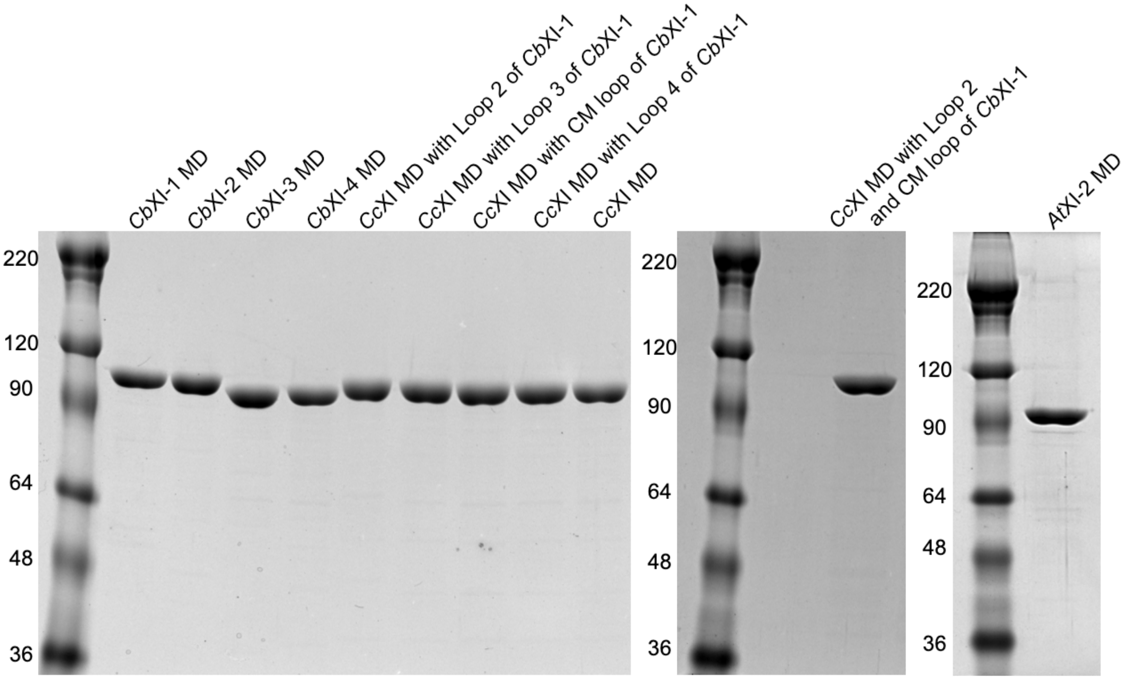

Figure S2. SDS-PAGE of purified myosins. Purified myosins were electrophoresed on 8% SDS-PAGE and stained with Coomassie Brilliant Blue. **Left**: *Cb*XI-1 MD, *Cb*XI-2 MD, *Cb*XI-3 MD, *Cb*XI-4 MD, *Cc*XI MD with Loop 2 of *Cb*XI-1, *Cc*XI MD with Loop 3 of *Cb*XI-1, *Cc*XI MD with CM loop of *Cb*XI-1, *Cc*XI MD with Loop 4 of *Cb*XI-1, and *Cc*XI MD were analyzed using 8% SDS-PAGE and stained with Coomassie Brilliant Blue. **Center**: *Cc*XI MD with Loop 2 and CM loop of *Cb*XI-1. **Right**: *At*XI-2 MD. The positions of molecular mass markers are indicated on the left (kDa).

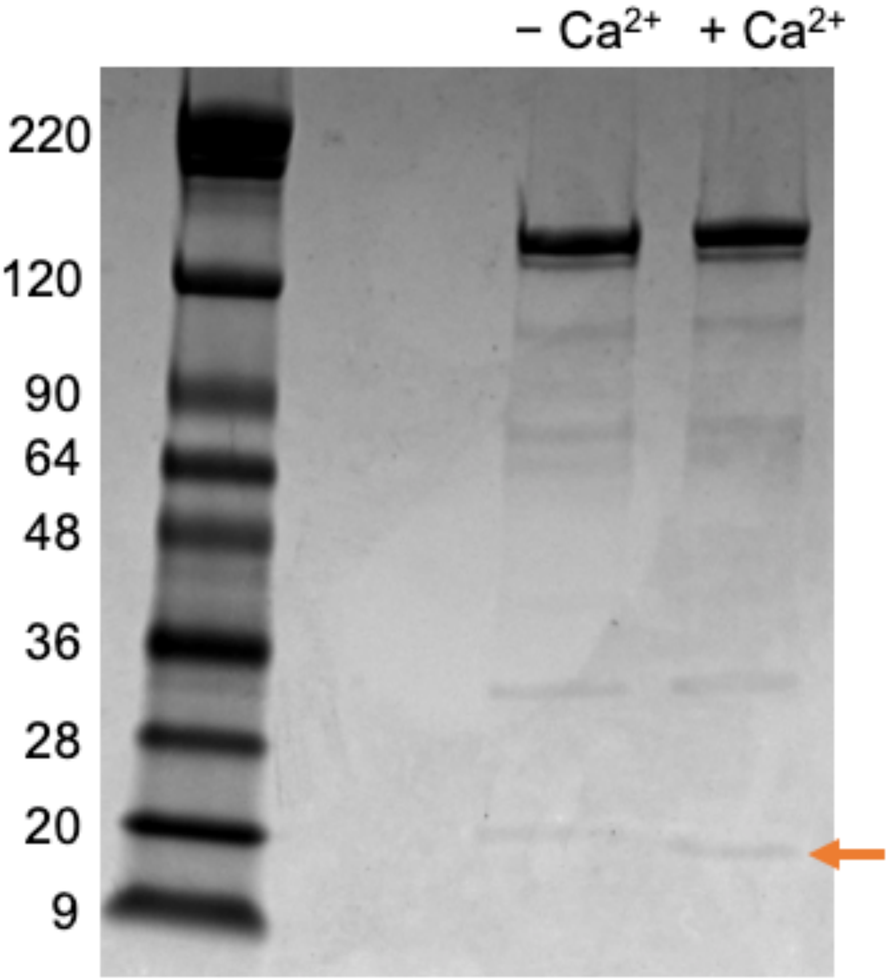

Figure S3. SDS-PAGE of purified chimeric *Cb*XI-1showing calmodulin binding. Purified chimeric *Cb*XI-1 was electrophoresed on 4–20% SDS-PAGE and stained with Coomassie Brilliant Blue. Before electrophoresis, SDS samples were supplemented with 10 mM EGTA (- Ca^{2+}) or 10 mM $CaCl_2$ (+ Ca^{2+}). The low-molecular-weight band indicated by the red arrow with mobility shifted by Ca^{2+} is considered to be the co-expressed *Arabidopsis* calmodulin. Note that Coomassie brilliant blue staining of calmodulin is weaker than that of myosin due to the differences in amino acid composition. The positions of molecular mass markers are indicated on the left (kDa).

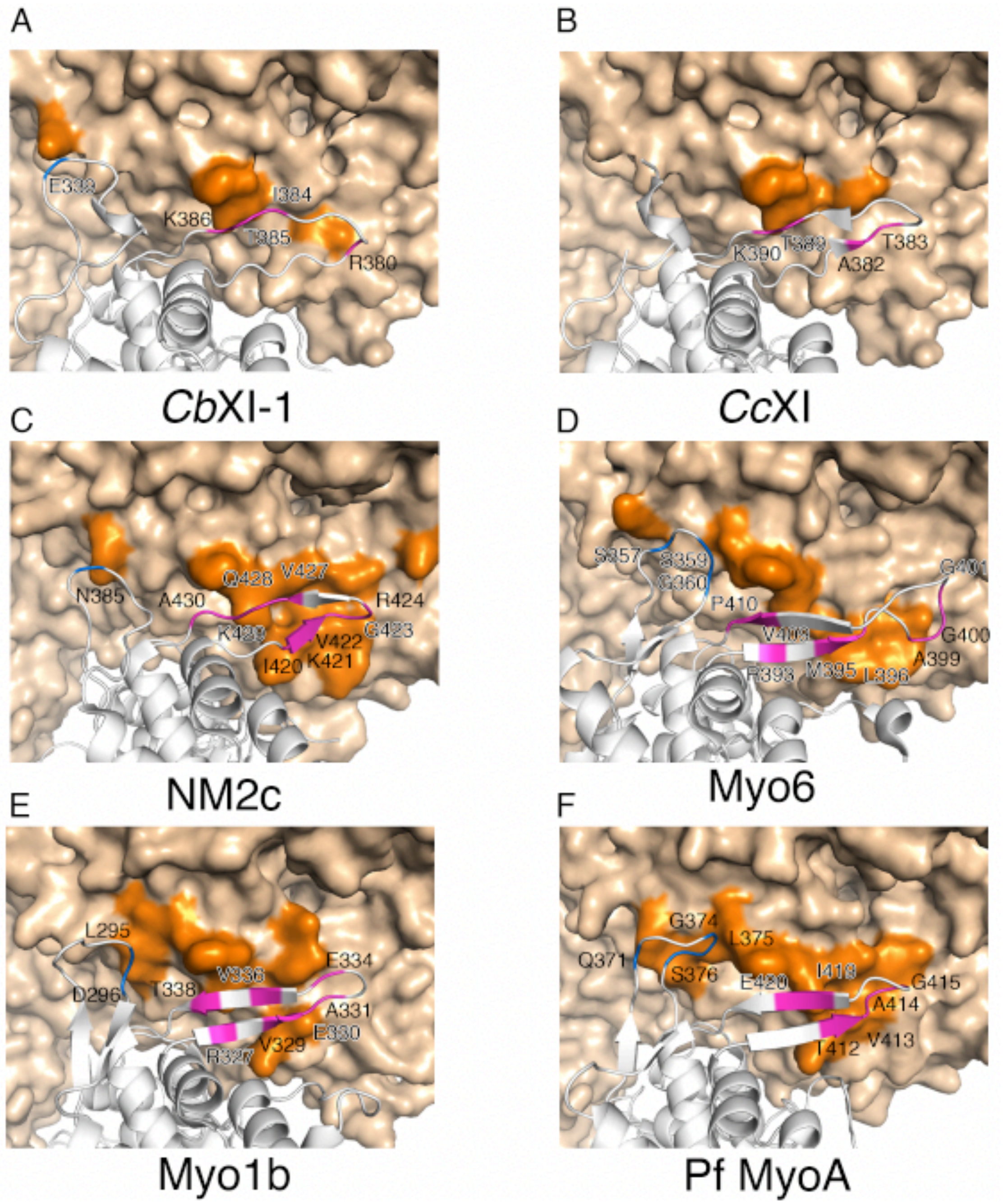

Figure S4. Interaction of CM loop and loop4 of various myosins with actin. The interactions of MDs (white) of *Cb*XI-1 (**A**, homology model built using PDB: 7KCH), *Cc*XI (**B**, PDB: 7KCH), NM2c (**C**, PDB: 5JLH), Myo6 (**D**, PDB: 6BNP), Myo1b (**E**, PDB: 6C1H), Pf MyoA (**F**, PDB: 7ALN) and actin (light orange) are shown as ribbon and surface representations. The residues in the actin, CM loop, and loop 4 within 4 Å between the actin and myosin are colored in orange, magenta, and blue, respectively.

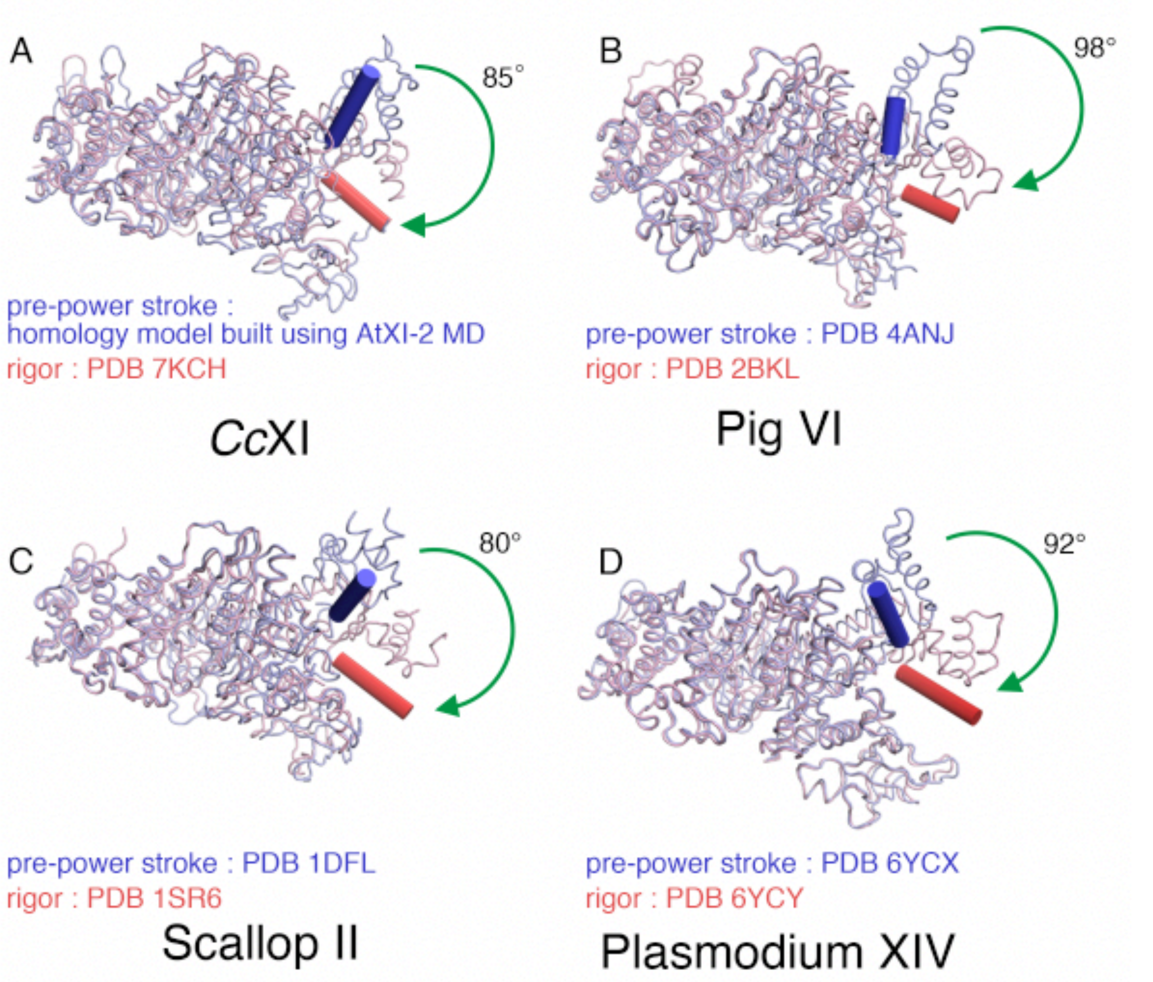

Figure S5. Structural comparison of pre-power stroke state and rigor state.
Angular changes in the lever arm (the last helices of converter subdomain shown as syringe) between the pre-power state (blue) and rigor (red) state for *Cc*XI (**A**), Pig myosin VI (**B**), Scallop myosin II (**C**), and Plasmodium myosin XIV (**D**) were examined by superimposing the structures of the two states for Upper 50k and Lower 50k subdomains. The converter subdomains are shown in darker colors.

Table S1. Analysis of the IQ motif sequences of myosins using Pfam database

Myosins	*E*-value of 1st IQ	*E*-value of 2nd IQ	*E*-value of 3rd IQ	*E*-value of 4th IQ	*E*-value of 5th IQ	*E*-value of 6th IQ	Calmodulin as light chains
Myosin XIs							
Cc XI	200	0.04	0.0017	1.9	0.06	5200	No[a]
Cb XI-1	90	4.6	4	160	0.051	3.6	No[b]
At XI-2	0.054	0.00052	0.0031	0.18	0.0000064	0.16	Yes[c, d]
At XI-A	12	0.42	13	1200	0.17	0.11	No[d]
At XI-D	730	0.006	12	3	0.0014	1.5	No[d]
At XI-F	0.017	0.039	0.0023	6.5	0.011	0.082	Yes[d]
At XI-I	63	0.0032	0.03	16	0.023	0.11	No[d]
At XI-J	460	0.0046	0.037	1.5	0.00031	1100	No[d]
Tobacco XI	0.0068	0.25	0.058	0.81	0.053	0.38	Yes[e]
Animal unconventional myosins							
Human IC	0.00083	0.00051	63				Yes[f]
Chick Va	0.0017	0.0009	0.00094	0.0047	0.00034	0.18	Yes[g]
Mouse Va	0.0013	0.0041	0.000021	0.00012	0.0002	0.17	Yes[h, i, j]
Pig VI	0.018						Yes[k]
Mouse VIIB	0.0013	0.000052	69	0.097	0.013		Yes[l]
Bovine X	0.003	0.0039	0.057				Yes[m, n]

IQ motif sequences of myosins were analyzed using Pfam database (http://pfam.xfam.org) (24). Most of the IQ motif sequences of unconventional myosins, in which calmodulin acts light chains (indicated by Yes), fall into the typical calmodulin-binding IQ motif, especially for the 1st IQ motif sequence: Pfam *E*-values for calmodulin-binding motif of these myosins are less than 0.1. On the other hand, *E*-values of unconventional myosins, in which calmodulin does not act as light chains (indicated by No), range from several tens to several hundreds, and this is especially true for the 1st IQ motif sequence.

[a](2), [b]this paper, [c](25), [d](3), [e](26), [f](27), [g](28), [h](29), [i](30), [j](31), [k](32), [l](33), [m](34), [n](35)

Table S2. Crystallographic data collection and refinement statistics for *At* XI-2 MD

**Data collection**	
Space group	$P2_12_12_1$
Cell dimensions	
a, *b*, *c* (Å)	58.28, 73.73, 187.96
α, β, γ (°)	90.0, 90.0, 90.0
Resolution (Å)	19.81-2.84 (3.01-2.84)*
R_{merge}	0.132 (0.840)
$I/\sigma I$	11.09 (2.10)
Completeness (%)	99.1 (98.0)
Redundancy	5.3 (5.6)
**Refinement**	
Resolution (Å)	19.81-2.84
No. reflections	104235
$R_{work/}$ R_{free} (%)	22.64/27.06
R.m.s deviations	
Bond lengths (Å)	0.002
Bond angles (º)	0.490
Ramachandran plot statistics (%)	
Favored regions	99.0
Allowed regions	1.0
Outliers	0.0

*Highest resolution shell is shown in parentheses.

Table S3. List of amino acids interacting between CM loop and loop 4 of myosins and actin

CM loop

Actin residues	*Cc* XI	*Cb* XI-1	*Pf* MyoA	Pig VI	*Hs* NM2c	Rat Ib
D27			T412<br>A414	M395	I420	R327
A28	A382		T412<br>V413	L396	I420<br>K421<br>V422	E330
P29		R380	A414<br>G415	A399	K421<br>V422<br>G423	
R30				A399<br>G400<br>G401	G423	
V32					V422<br>G423	
D58					R424	
P335	T389	I384<br>T385	E420<br>I419	V409<br>P410	V427<br>A430	V336<br>T338
E336	K390	K386	I419<br>T412	M395<br>R393<br>V409	Q428<br>K429	V329<br>T338
K338						E334
Y339	T383				V422	A331<br>E334
I343				R393		

Loop 4

Actin residues	*Cc* XI	*Cb* XI-1	*Pf* MyoA	Pig VI	*Hs* NM2c	Rat Ib
R149			S376			D296
K328		E339				
K330			Q371<br>S376	S357	N385	
I331			S376			L295
I332						L295
A333			G374<br>L375	S359<br>G360		L295
P334			L375	S359<br>G360		
P335				S359		

Amino acid residues of loop 4 and CM loop with a distance of 4 Å or less from actin are shown. The amino acid residues of actin are based on the sequence of alpha skeletal muscle, *Gallus gallus* (PDBID: 7KCH). *Cc* XI: (36) (PDBID: 7KCH), Rat Ib: (37) (PDBID: 6C1H), *Cb* XI-1: (this paper), *Pf* MyoA, *Plasmodium falciparum* myosin A (38) (PDBID: 7ALN), Pig myosin VI: (39) (PDBID: 6BNP), *Hs* NM2c: *Homo sapiens* non-muscle myosin 2c (40) (PDBID: 5JLH)

Table S4. Expression levels of *Cb*XIs in *Chara braunii*

	RNA-Seq (FPKM)			
	**Whole plant**	**oogonia**	**antheridia**	**zygote**
*Cb*XI-1	28.57	22.96	14.62	0.70
*Cb*XI-2	3.96	15.17	6.13	13.59
*Cb*XI-3	2.77	12.10	5.18	13.70
*Cb*XI-4	3.95	8.42	8.33	2.94

data from (1)

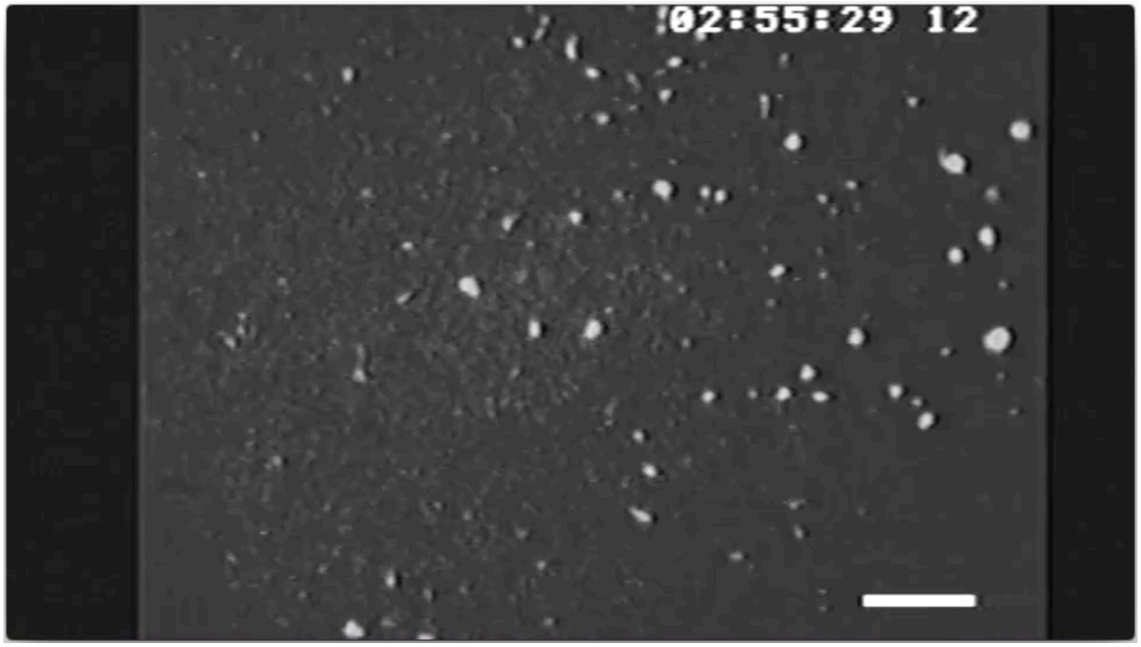

Movies S1. *in vitro* motility assay of chimeric *Cb*XI-1 (real time).

References for Supplementary Information

1. T. Nishiyama *et al.*, The Chara Genome: Secondary Complexity and Implications for Plant Terrestrialization. *Cell* **174**, 448-464 e424 (2018).
2. K. Ito *et al.*, Kinetic mechanism of the fastest motor protein, Chara myosin. *J Biol Chem* **282**, 19534-19545 (2007).
3. T. Haraguchi *et al.*, Functional Diversity of Class XI Myosins in Arabidopsis thaliana. *Plant Cell Physiol* **59**, 2268-2277 (2018).
4. K. Ito, Y. Yamaguchi, K. Yanase, Y. Ichikawa, K. Yamamoto, Unique charge distribution in surface loops confers high velocity on the fast motor protein Chara myosin. *Proc Natl Acad Sci U S A* **106**, 21585-21590 (2009).
5. T. Haraguchi *et al.*, Molecular Characterization and Subcellular Localization of Arabidopsis Class VIII Myosin, ATM1. *J Biol Chem* **289**, 12343-12355 (2014).
6. W. Kabsch, Xds. *Acta Crystallogr D Biol Crystallogr* **66**, 125-132 (2010).
7. A. J. McCoy *et al.*, Phaser crystallographic software. *J Appl Crystallogr* **40**, 658-674 (2007).
8. P. Emsley, K. Cowtan, Coot: model-building tools for molecular graphics. *Acta Crystallogr D Biol Crystallogr* **60**, 2126-2132 (2004).
9. P. D. Adams *et al.*, PHENIX: a comprehensive Python-based system for macromolecular structure solution. *Acta Crystallogr D Biol Crystallogr* **66**, 213-221 (2010).
10. S. C. Lovell *et al.*, Structure validation by Calpha geometry: phi,psi and Cbeta deviation. *Proteins* **50**, 437-450 (2003).
11. B. Webb, A. Sali, Comparative Protein Structure Modeling Using MODELLER. *Curr Protoc Bioinformatics* **54**, 5 6 1-5 6 37 (2016).
12. F.-W. Li *et al.*, Fern genomes elucidate land plant evolution and cyanobacterial symbioses. *Nature Plants* **4**, 460-472 (2018).
13. J. L. Bowman *et al.*, Insights into Land Plant Evolution Garnered from the Marchantia polymorpha Genome. *Cell* **171**, 287-304.e215 (2017).
14. F.-W. Li *et al.*, Anthoceros genomes illuminate the origin of land plants and the unique biology of hornworts. *Nature Plants* **6**, 259-272 (2020).
15. S. Cheng *et al.*, Genomes of Subaerial Zygnematophyceae Provide Insights into Land Plant Evolution. *Cell* **179**, 1057-1067.e1014 (2019).
16. K. Katoh, D. M. Standley, MAFFT multiple sequence alignment software version 7: improvements in performance and usability. *Mol Biol Evol* **30**, 772-780 (2013).
17. W. P. Maddison, D. R. Maddison, Mesquite: a modular system for evolutionary analysis. *Version 3.61* **http://www.mesquiteproject.org** (2019).
18. A. Stamatakis, RAxML version 8: a tool for phylogenetic analysis and post-analysis of large phylogenies. *Bioinformatics* **30**, 1312-1313 (2014).
19. J. Felsenstein, PHYLIP (Phylogeny Inference Package) version 3.697. (Distributed by the author. Department of Genome Sciences, University of Washington, Seattle.). (2017).
20. S. F. Altschul *et al.*, Gapped BLAST and PSI-BLAST: a new generation of protein database search programs. *Nucleic Acids Res* **25**, 3389-3402 (1997).
21. G. Robertson *et al.*, De novo assembly and analysis of RNA-seq data. *Nature Methods* **7**, 909-912 (2010).

22. M. Stanke *et al.*, AUGUSTUS: ab initio prediction of alternative transcripts. *Nucleic Acids Research* **34**, W435-W439 (2006).
23. E. Lee *et al.*, Web Apollo: a web-based genomic annotation editing platform. *Genome Biology* **14**, R93 (2013).
24. A. Bateman *et al.*, The Pfam protein families database. *Nucleic Acids Research* **32**, D138-D141 (2004).
25. M. Tominaga *et al.*, Cytoplasmic streaming velocity as a plant size determinant. *Dev Cell* **27**, 345-352 (2013).
26. M. Tominaga *et al.*, Higher plant myosin XI moves processively on actin with 35 nm steps at high velocity. *EMBO J* **22**, 1263-1272 (2003).
27. M. El_Mezgueldi, N. Tang, S. S. Rosenfeld, E. M. Ostap, The kinetic mechanism of Myo1e (human myosin-IC). *J. Biol. Chem.* **277**, 21514-21521 (2002).
28. E. M. De_La_Cruz, A. L. Wells, S. S. Rosenfeld, E. M. Ostap, H. L. Sweeney, The kinetic mechanism of myosin V. *Proc. Natl. Acad. Sci. USA* **96**, 13726-13731 (1999).
29. K. Homma, J. Saito, R. Ikebe, M. Ikebe, Ca(2+)-dependent regulation of the motor activity of myosin V. *J. Biol. Chem.* **275**, 34766-34771 (2000).
30. F. Wang *et al.*, Effect of ADP and ionic strength on the kinetic and motile properties of recombinant mouse myosin V. *J Biol Chem* **275**, 4329 - 4335 (2000).
31. K. M. Trybus, E. Krementsova, Y. Freyzon, Kinetic characterization of a monomeric unconventional myosin V construct. *J. Biol. Chem.* **274**, 27448-27456 (1999).
32. A. L. Wells *et al.*, Myosin VI is an actin-based motor that moves backwards. *Nature* **401**, 505-508 (1999).
33. A. Henn, E. M. De La Cruz, Vertebrate myosin VIIb is a high duty ratio motor adapted for generating and maintaining tension. *J Biol Chem* **280**, 39665-39676 (2005).
34. K. Homma, M. Ikebe, Myosin x is a high duty ratio motor. *J Biol Chem* **280**, 29381 - 29391 (2005).
35. M. Kov·cs, F. Wang, J. R. Sellers, Mechanism of action of myosin X, a membrane-associated molecular motor. *J Biol Chem* **280**, 15071 - 15083 (2005).
36. P. V. Ruijgrok *et al.*, Optical control of fast and processive engineered myosins in vitro and in living cells. *Nat Chem Biol* 10.1038/s41589-021-00740-7 (2021).
37. A. Mentes *et al.*, High-resolution cryo-EM structures of actin-bound myosin states reveal the mechanism of myosin force sensing. *Proc Natl Acad Sci U S A* **115**, 1292-1297 (2018).
38. J. Robert-Paganin *et al.*, The actomyosin interface contains an evolutionary conserved core and an ancillary interface involved in specificity. *Nat Commun* **12**, 1892 (2021).
39. P. S. Gurel *et al.*, Cryo-EM structures reveal specialization at the myosin VI-actin interface and a mechanism of force sensitivity. *Elife* **6** (2017).
40. J. von der Ecken, S. M. Heissler, S. Pathan-Chhatbar, D. J. Manstein, S. Raunser, Cryo-EM structure of a human cytoplasmic actomyosin complex at near-atomic resolution. *Nature* **534**, 724-728 (2016).
